# A Factor Analysis Perspective on Linear Regression in the ‘More Predictors than Samples’ Case

**DOI:** 10.3390/e23081012

**Published:** 2021-08-03

**Authors:** Sebastian Ciobanu, Liviu Ciortuz

**Affiliations:** Faculty of Computer Science, Alexandru Ioan Cuza University of Iaşi, 700506 Iaşi, Romania

**Keywords:** more predictors than samples, linear regression, factor analysis, semisupervised regression, missing data

## Abstract

Linear regression (LR) is a core model in supervised machine learning performing a regression task. One can fit this model using either an analytic/closed-form formula or an iterative algorithm. Fitting it via the analytic formula becomes a problem when the number of predictors is greater than the number of samples because the closed-form solution contains a matrix inverse that is not defined when having more predictors than samples. The standard approach to solve this issue is using the Moore–Penrose inverse or the L2 regularization. We propose another solution starting from a machine learning model that, this time, is used in unsupervised learning performing a dimensionality reduction task or just a density estimation one—factor analysis (FA)—with one-dimensional latent space. The density estimation task represents our focus since, in this case, it can fit a Gaussian distribution even if the dimensionality of the data is greater than the number of samples; hence, we obtain this advantage when creating the supervised counterpart of factor analysis, which is linked to linear regression. We also create its semisupervised counterpart and then extend it to be usable with missing data. We prove an equivalence to linear regression and create experiments for each extension of the factor analysis model. The resulting algorithms are either a closed-form solution or an expectation–maximization (EM) algorithm. The latter is linked to information theory by optimizing a function containing a Kullback–Leibler (KL) divergence or the entropy of a random variable.

## 1. Introduction

In machine learning, models can be grouped into two categories: probabilistic and nonprobabilistic. Probabilistic models can be classified as generative and discriminative [1]. Examples of classic generative models are naive Bayes and Gaussian mixture models (GMM). Examples of classic discriminative models are linear regression (LR) and logistic regression. The key difference is whether they model the joint probability of the input and the output—generative models—or they just model the conditional probability of the output given the input—discriminative models. For a classification or a regression task, one may argue that what you need is just a discriminative model, but the generative models have their advantages: they can sometimes handle missing data, can easily generate new data, can be extended to be unsupervised or semisupervised, etc. ([2] p. 268).

As one may notice, there are generative models for unsupervised learning that have counterparts in supervised learning, even though this is not widely discussed in the literature. One such example is the GMM ([2] p. 339) with its counterpart, the Gaussian joint Bayes model ([2] p. 102), also known as quadratic discriminant analysis. Their training/fitting algorithms are similar, as one may notice, for example, in [3,4]:

- for Gaussian joint Bayes:
  $$\pi_{j}=\frac{1}{n}\sum_{i=1}^{n}1_{\left\{z_{i}=j\right\}}$$
  $$\mu_{j}=\frac{\sum_{i=1}^{n}1_{\left\{z_{i}=j\right\}}x_{i}}{\sum_{i=1}^{n}1_{\left\{z_{i}=j\right\}}}$$
  $$\Sigma_{j}=\frac{\sum_{i=1}^{n}1_{\left\{z_{i}=j\right\}}\left(x_{i}-\mu_{j}\right){\left(x_{i}-\mu_{j}\right)}^{⊤}}{\sum_{i=1}^{n}1_{\left\{z_{i}=j\right\}}}$$
  where $x_{i}$ is an input observation, ($\pi_{j},\mu_{j},\Sigma_{j}$) are the parameters of a GMM, observable $z_{i}$ is the class index corresponding to $x_{i}$, *j* is a class index, and $1_{\left\{z_{i}=j\right\}}$ is the indicator function, which returns 1 if the condition $z_{i}=j$ is true and 0 otherwise.
- for expectation–maximization (EM) for the GMM, which optimizes a function concerning a Kullback–Leibler (KL) divergence or the entropy of a random variable—check Appendix A for these details—:
  E step:
  $$\begin{array}{cc} w_{ij} & =p(z_{i}=j|x_{i};\pi^{\prime },\mu^{\prime },\Sigma^{\prime })\\ \; & =\frac{p\left(x_{i}|z_{i}=j;\mu^{\prime },\Sigma^{\prime }\right)p\left(z_{i}=j;\pi^{\prime }\right)}{\sum_{l=1}^{K}p\left(x_{i}|z_{i}=l;\mu^{\prime },\Sigma^{\prime }\right)p\left(z_{i}=l;\pi^{\prime }\right)} \end{array}$$
  M step:
  $$\pi_{j}=\frac{1}{n}\sum_{i=1}^{n}w_{ij}$$
  $$\mu_{j}=\frac{\sum_{i=1}^{n}w_{ij}x_{i}}{\sum_{i=1}^{n}w_{ij}}$$
  $$\Sigma_{j}=\frac{\sum_{i=1}^{n}w_{ij}\left(x_{i}-\mu_{j}\right){\left(x_{i}-\mu_{j}\right)}^{⊤}}{\sum_{i=1}^{n}w_{ij}}$$
  where $x_{i}$ is an input observation, ($\pi_{j},\mu_{j},\Sigma_{j}$) are the parameters of a GMM, unobservable $z_{i}$ is the cluster index corresponding to $x_{i}$, *j* is a cluster index, and $w_{ij}$ is the probability that $x_{i}$ belongs to cluster *j*.

This similarity between GMM and Gaussian joint Bayes is intriguing; hence, we decided to further explore this aspect but starting from other supervised–unsupervised counterparts. As a result, we changed the root model into factor analysis (FA) [5] ([2] p. 381), which is normally used for dimensionality reduction or for density estimation when the dimensionality of the data is greater than the number of samples. Factor analysis is a Gaussian generative model used in unsupervised learning. We aimed at creating its supervised counterpart in order to handle a regression task and then exploit it as much as possible.

After creating the supervised counterpart, we proved a significant property, namely that linear regression is equivalent to (supervised) factor analysis—with one-dimensional latent space—when no constraints are imposed on the covariance matrices.

A linear regression model can be fitted via a closed-form solution or an iterative algorithm. When the number of predictors is greater than the number of samples, there is no closed-form solution. There are other solutions to this problem, as we will see.

We were at the point where we knew that factor analysis was linked to linear regression and that it could be used when the number of samples was lower than the dimensionality of the data—from now on, this is denoted as D>>n or n<<D. As a result, we shifted our focus from solely exploiting the factor analysis model to highlighting novel linear regression versions applicable in the D>>n regime—linear regression being a widely known and used model—:

- linear regression when D>>n,
- semisupervised linear regression when D>>n,
- (semisupervised) linear regression when D>>n with missing data.

The structure of this paper is as follows. In Section 2, we include some theoretical background to enhance the readability of this paper. Section 3 contains related work. In Section 4, we include the models we proposed, starting from factor analysis. Section 5 contains experiments using the proposed models. In Section 6, we conclude the paper and show future directions.

We include the full algorithms in the appendices in a pseudocode format, two of them being instances of the expectation–maximization schema.

## 2. Theoretical Background

We started our analysis from two core models in machine learning: linear regression and factor analysis. We will discuss the aspects of those two models that are relevant to understanding the next sections of this paper.

### 2.1. Linear Regression

**Proposition** **1.** *Let $\left\{\left(x^{\left(i\right)},y^{\left(i\right)}\right)|x^{\left(i\right)}\in R^{D\times 1},y^{\left(i\right)}\in R,i\in \left\{1,\ldots ,n\right\}\right\}$ be a data set where D is the dimensionality of the input data, $\left\{x^{\left(i\right)}|i\in \left\{1,\ldots ,n\right\}\right\}$ is the input, and $\left\{y^{\left(i\right)}|i\in \left\{1,\ldots ,n\right\}\right\}$ is the output. The linear regression model is as follows:*

$$Y^{\left(i\right)}=wx^{\left(i\right)}+b+\epsilon^{\left(i\right)}$$

where $Y^{\left(i\right)}$ is a random variable corresponding to $y^{\left(i\right)}$, $\epsilon^{\left(i\right)}∼N\left(0,\sigma^{2}\right)$, $\sigma \in R_{+}^{*}$, $w\in R^{1\times D}$, b∈R. Then, the parameters w and b can be estimated via maximum likelihood as follows:

(1) $$\begin{array}{cc} {\hat{w}}_{\mathrm{LR}} & =\left(n\bar{y}{\bar{x}}^{⊤}-YX^{⊤}\right){\left(n\bar{x}{\bar{x}}^{⊤}-XX^{⊤}\right)}^{-1} \end{array}$$

(2) $$\begin{array}{cc} {\hat{b}}_{\mathrm{LR}} & =\bar{y}-{\hat{w}}_{\mathrm{LR}}\bar{x}, \end{array}$$

*or, equivalently, as follows:*

(3) $$\begin{array}{c} \left[\begin{array}{c} {\hat{w}}_{\mathrm{LR}}^{⊤}\\ {\hat{b}}_{\mathrm{LR}} \end{array}\right]={\left(\tilde{X}{\tilde{X}}^{⊤}\right)}^{-1}\tilde{X}Y^{⊤} \end{array}$$

*where $\bar{x}=\frac{x^{\left(1\right)}+\cdots +x^{\left(n\right)}}{n}$, $\bar{y}=\frac{y^{\left(1\right)}+\cdots +y^{\left(n\right)}}{n}$,*

*$X=\left[\begin{array}{ccc} x^{\left(1\right)} & \ldots & x^{\left(n\right)} \end{array}\right]\in R^{D\times n},$$\tilde{X}=\left[\begin{array}{ccc} {\tilde{x}}^{\left(1\right)} & \ldots & {\tilde{x}}^{\left(n\right)} \end{array}\right]\in R^{(D+1)\times n},$*

*${\tilde{x}}^{\left(i\right)}=\left[\begin{array}{c} x^{\left(i\right)}\\ 1 \end{array}\right]=\left[\begin{array}{c} x_{1}^{\left(i\right)}\\ \vdots \\ x_{D}^{\left(i\right)}\\ 1 \end{array}\right]\in R^{D+1},$ and $Y=\left[\begin{array}{ccc} y^{\left(1\right)} & \ldots & y^{\left(n\right)} \end{array}\right]\in R^{1\times n}$.*

A potential problem with Equation (3) is when $\tilde{X}{\tilde{X}}^{⊤}$ is not invertible. Such case arises when D+1>n, i.e., there are more predictors than samples. Two standard solutions to this problem are the following:

- Let $A\in R^{a\times b}$ be a matrix. Then, the Moore–Penrose inverse of *A* can be defined as $A^{+}=\lim_{\alpha \to 0^{+}}{\left(A^{⊤}A+\alpha I\right)}^{-1}A^{⊤}$, which, algorithmically, is computed via the singular value decomposition of *A* ([6] Section 2.9). One may notice that the matrix ${\left(\tilde{X}{\tilde{X}}^{⊤}\right)}^{-1}\tilde{X}$ from Equation (3) is just ${\left({\tilde{X}}^{⊤}\right)}^{+}$ when $\tilde{X}{\tilde{X}}^{⊤}$ is invertible. When it is not, the solution is to replace the matrix ${\left(\tilde{X}{\tilde{X}}^{⊤}\right)}^{-1}\tilde{X}$ from Equation (3) with ${\left({\tilde{X}}^{⊤}\right)}^{+}$.
- L2 regularization, which results in ridge regression ([2] p. 225). The matrix ${\left(\tilde{X}{\tilde{X}}^{⊤}\right)}^{-1}\tilde{X}$ from Equation (3) is replaced with ${\left(\tilde{X}{\tilde{X}}^{⊤}+\left[\begin{array}{cccc} \alpha & \ldots & 0 & 0\\ \vdots & \ddots & \vdots & \vdots \\ 0 & \ldots & \alpha & 0\\ 0 & \ldots & 0 & 0 \end{array}\right]\right)}^{-1}\tilde{X}$, with α>0; the bigger the α, the more regularization we add to the model, i.e., move away from overfitting. From this point of view, the first solution using the Moore–Penrose inverse can be interpreted as achieving the asymptotically lowest L2 regularization.

### 2.2. Factor Analysis

The formulas stated in the conclusion of the following Proposition were proved in [5] and are relevant for the factor analysis algorithm—although the matrix Ψ is considered as being diagonal there, the formulas stay the same even if Ψ is not diagonal.

**Proposition** **2.** *Let us consider the following factor analysis model:*

*z∼N(0,I)-latent variable, $z\in R^{d\times 1}$*

*x|z∼N(μ+Λz,Ψ), $x\in R^{D\times 1},\mu \in R^{D\times 1},\Lambda \in R^{D\times d},\;\mathrm{and}\;\Psi \in R^{D\times D}$ a diagonal matrix. Then:*

$\left[\begin{array}{c} x\\ z \end{array}\right]∼N\left(\left[\begin{array}{c} \mu \\ 0 \end{array}\right],\left[\begin{array}{cc} \Lambda \Lambda^{⊤} & \Lambda \\ \Lambda^{⊤} & I \end{array}\right]\right)$

$x∼N(\mu ,\Lambda \Lambda^{⊤}+\Psi )$

$z|x∼N(\Lambda^{⊤}{\left(\Lambda \Lambda^{⊤}+\Psi \right)}^{-1}\left(x-\mu \right),I-\Lambda^{⊤}{\left(\Lambda \Lambda^{⊤}+\Psi \right)}^{-1}\Lambda ).$

A factor analysis model can be fitted via an EM algorithm using the maximum likelihood estimation (MLE) principle, and factor analysis can be used as a density estimation technique when the dimensionality of the data is greater than the number of samples [5].

For the algorithms we developed, we will let $z∼N(\mu_{z},\Sigma_{z})$ and not z∼N(0,I), because *z* becomes observed data, and we want to learn its parameters, and not impose something unrealistic like z∼N(0,I). This generalization leads to the following result.

**Proposition** **3.** *Let us consider the following linear Gaussian system:*

*$z∼N(\mu_{z},\Sigma_{z})$-latent variable, $z\in R^{d\times 1}$, $\mu_{z}\in R^{d\times 1}$, $\Sigma_{z}\in R^{d\times d}$ a symmetric and positive definite matrix,*

x|z∼N(μ+Λz,Ψ), $x\in R^{D\times 1},\mu \in R^{D\times 1},\Lambda \in R^{D\times d}$, and $\Psi \in R^{D\times D}$ a symmetric and positive definite matrix. (If *Ψ* is a diagonal matrix, then we say that we are in the FA case. If *Ψ* is a scalar matrix, Ψ=ηI, $\eta \in R_{+}^{*}$, then we are in the probabilistic principal component analysis (PPCA) case [7]. If *Ψ* is any symmetric and positive definite matrix, then we say we are in the unconstrained factor analysis (UncFA) case. If the first two terms are standard (FA and PPCA), the third one is proposed by us—UncFA.) Then:

(4) $$\begin{array}{c} \left[\begin{array}{c} x\\ z \end{array}\right]∼N\left(\left[\begin{array}{c} \mu +\Lambda \mu_{z}\\ \mu_{z} \end{array}\right],\left[\begin{array}{cc} \Lambda \Sigma_{z}\Lambda^{⊤}+\Psi & \Lambda \Sigma_{z}\\ {\left(\Lambda \Sigma_{z}\right)}^{⊤} & \Sigma_{z} \end{array}\right]\right)\\ x∼N(\mu +\Lambda \mu_{z},\Lambda \Sigma_{z}\Lambda^{⊤}+\Psi )\\ z|x∼N(\mu_{z}+\Sigma_{z}\Lambda^{⊤}{\left(\Lambda \Sigma_{z}\Lambda^{⊤}+\Psi \right)}^{-1}\left(x-\mu -\Lambda \mu_{z}\right),\\ \;\Sigma_{z}-\Sigma_{z}\Lambda^{⊤}{\left(\Lambda \Sigma_{z}\Lambda^{⊤}+\Psi \right)}^{-1}\Lambda \Sigma_{z}^{⊤}). \end{array}$$

The proof can be found in ([8] pp. 9–11).

## 3. Related Work

Although factor analysis is widely used for dimensionality reduction, its supervised counterpart is, to the best of our knowledge, not present in the literature. What is present is a model called supervised principal component analysis or latent factor regression ([2] p. 405). The idea is that not only the input, for a regression task, is generated by a latent variable, as one applies factor analysis to replace the input in the problem with a low dimensional embedding, but also the output. The key idea is that the purpose of supervised principal component analysis is still dimensionality reduction and not at all regression, which is where we want to push the factor analysis model.

There is also a term called linear Gaussian system ([2] p. 119). This was already presented in Section 2, and it generalizes the factor analysis generative process by considering that *z* has a learnable mean and covariance matrix, but it does not go further.

Factor analysis is strongly related to principal component analysis (PCA) [9] because by imposing a certain constraint in factor analysis, we get a model called probabilistic principal component analysis [7] that can be fitted using a closed-form solution, which, in an asymptotic case, is also the solution for PCA. Probabilistic PCA can be kernelized using a model called the Gaussian process latent variable model (GPLVM) [10]. This model also has supervised counterparts [11], but, as in the case of FA, the supervised extension targets dimensionality reduction, and the idea is similar to the one in supervised PCA.

## 4. Proposed Models

In this section, we propose three models starting from the FA model, each in a new subsection: simple-supervised factor analysis (S2.FA), simple-semisupervised factor analysis (S3.FA), and missing simple-semisupervised factor analysis (MS3.FA). While S2.FA is applicable in the supervised case, regression, S3.FA is meant to be used in a semisupervised context. MS3.FA handles missing input data in a (semi)supervised scenario.

One important remark that will not be restated in this paper is that all the models (FA, S2.FA, S3.FA, MS3.FA) are fitted by maximizing the likelihood (the MLE principle) of the observed data. Another important observation is regarding the names of our proposed algorithms: the algorithms are called “simple-”—S2 = simple-supervised; S3 = simple-semisupervised; MS3 = missing simple-semisupervised—not only because they constitute a simple adaptation of the factor analysis model, but mostly because we created an adaptation of the (simple-)supervised FA model called (simple-)supervised PPCA, and we did not want this model to be confused with the already existing supervised PCA model in the literature. Simple-supervised probabilistic principal component analysis (S2.PPCA) is not discussed in this paper, but it is implemented and usable in the R package that we developed (https://github.com/aciobanusebi/s2fa; accessed on 31 July 2021) along with other undiscussed but related models: Simple-semisupervised unconstrained factor analysis (S3.UncFA), Simple-semisupervised probabilistic principal component analysis (S3.PPCA), Missing simple-semisupervised unconstrained factor analysis (MS3.UncFA), and Missing simple-semisupervised probabilistic principal component analysis (MS3.PPCA).

### 4.1. The S2.FA Model

The core of this subsection regards the S2.FA model, but in order to link it with LR, we need to also introduce a similar model to S2.FA: S2.UncFA. This link will make S2.FA a good candidate for replacing LR when D>>n. These three ideas—S2.FA and S2.UncFA, S2.UncFA-LR link, and replacing LR via S2.FA—will be expanded below.

#### 4.1.1. The S2.FA Model. S2.FA and S2.UncFA

The first model that we propose is called simple-supervised factor analysis. It is a linear Gaussian system with slight changes:

$z∼N(\mu_{z},\sigma_{z}^{2})$-observed variable, z∈R, $\mu_{z}\in R$, $\sigma_{z}\in R_{+}^{*}$

x|z∼N(μ+Λz,Ψ), $x\in R^{D\times 1},\mu \in R^{D\times 1},\Lambda \in R^{D\times 1}$, and $\Psi \in R^{D\times D}$ a diagonal matrix.

If we do not impose the constraint of Ψ being diagonal, we arrive at the simple-supervised unconstrained factor analysis (S2.UncFA):

$z∼N(\mu_{z},\sigma_{z}^{2})$-observed variable, z∈R, $\mu_{z}\in R$, $\sigma_{z}\in R_{+}^{*}$

x|z∼N(μ+Λz,Ψ), $x\in R^{D\times 1},\mu \in R^{D\times 1},\Lambda \in R^{D\times 1}$, and $\Psi \in R^{D\times D}$ a symmetric and positive definite matrix.

In contrast with the factor analysis model, which is fitted via an EM algorithm, S2.UncFA and S2.FA are fitted via analytic formulas (see Propositions 4 and 5).

**Proposition** **4.** *Let $\left\{\left(x^{\left(i\right)},z^{\left(i\right)}\right)|x^{\left(i\right)}\in R^{D\times 1},z^{\left(i\right)}\in R^{d\times 1},i\in \left\{1,\ldots ,n\right\}\right\}$ be a data set where D is the dimensionality of the input data, d is the dimensionality of the output data (although in the context of this paper d=1, we decided to expose more general results—d≥1—in order for the reader to gain more insight; this is the reason why we write $\Sigma_{z}$ and not just $\sigma_{z}^{2}$, or ${z^{\left(i\right)}}^{⊤}$ and not just $z^{\left(i\right)}$, or ${\bar{z}}^{⊤}$ and not just $\bar{z}$, etc.), $\left\{x^{\left(i\right)}|i\in \left\{1,\ldots ,n\right\}\right\}$ is the input, and $\left\{z^{\left(i\right)}|i\in \left\{1,\ldots ,n\right\}\right\}$ is the output. We suppose that the data was generated as follows:*

*$z^{\left(i\right)}∼N\left(\mu_{z},\Sigma_{z}\right)$, $z^{\left(i\right)}\in R^{d\times 1}$, $\mu_{z}\in R^{d\times 1}$, $\Sigma_{z}\in R^{d\times d}$ a symmetric and positive definite matrix and*

*$x^{\left(i\right)}|z^{\left(i\right)}∼N\left(\mu +\Lambda z,\Psi \right)$, $x^{\left(i\right)}\in R^{D\times 1},\mu \in R^{D\times 1},\Lambda \in R^{D\times d}$, while $\Psi \in R^{D\times D}$ is a symmetric and positive definite matrix.*

*Then, the parameters in the S2.UncFA algorithm (training phase) can be estimated via maximum likelihood using the following closed-form formulas:*

(5) $$\begin{array}{cc} {\hat{\mu }}_{z} & =\frac{\sum_{i=1}^{n}z^{\left(i\right)}}{n} \end{array}$$

(6) $$\begin{array}{cc} {\hat{\Sigma }}_{z} & =\frac{\sum_{i=1}^{n}\left(z^{\left(i\right)}-{\hat{\mu }}_{z}\right){\left(z^{\left(i\right)}-{\hat{\mu }}_{z}\right)}^{⊤}}{n} \end{array}$$

(7) $$\begin{array}{cc} \hat{\Lambda } & =\left(n\bar{x}{\bar{z}}^{⊤}-\sum_{i=1}^{n}x^{\left(i\right)}{z^{\left(i\right)}}^{⊤}\right){\left(n\bar{z}{\bar{z}}^{⊤}-\sum_{i=1}^{n}z^{\left(i\right)}{z^{\left(i\right)}}^{⊤}\right)}^{-1} \end{array}$$

(8) $$\begin{array}{cc} \hat{\mu } & =\bar{x}-\hat{\Lambda }\bar{z} \end{array}$$

(9) $$\begin{array}{cc} \hat{\Psi } & =\frac{\sum_{i=1}^{n}\left(x^{\left(i\right)}-\hat{\mu }-\Lambda z^{\left(i\right)}\right){\left(x^{\left(i\right)}-\hat{\mu }-\Lambda z^{\left(i\right)}\right)}^{⊤}}{n} \end{array}$$

*where $\bar{x}=\frac{\sum_{i=1}^{n}x^{\left(i\right)}}{n}$ and $\bar{z}={\hat{\mu }}_{z}$. For the testing/prediction phase, one uses the formula for z|x from (4).*

For more elaborate notations and the proof, see [8] pp. 13–17.

**Proposition** **5.** *[We will denote the parameter $\hat{\Psi }$ in (9) as ${\hat{\Psi }}_{S2.\mathrm{UncFA}}$.]*

*For the S2.FA algorithm, (9) is replaced by*

(10) $$\hat{\Psi }=\mathrm{diag}\left({\hat{\Psi }}_{S2.\mathrm{UncFA}}\right)$$

*where “diag” takes the diagonal of a matrix and returns the corresponding diagonal matrix.*

The proof of Equation (10) is relatively simple, and we skip it for brevity. It can be found in [8] pp. 21–23.

For the step-by-step S2.FA algorithm and also for the matrix form of the algorithm, see Appendix B.

#### 4.1.2. The S2.FA Model. The Link between LR and S2.UncFA

Linear regression and S2.UncFA have the same prediction function after fitting, as we claim and prove below.

**Proposition** **6.** *Let $\left\{\left(x^{\left(i\right)},z^{\left(i\right)}\right)|x^{\left(i\right)}\in R^{D\times 1},z^{\left(i\right)}\in R^{d},i\in \left\{1,\ldots ,n\right\}\right\}$ be a data set where D is the dimensionality of the input data, d is the dimensionality of the output data (The same observation as earlier: in the context of this paper, only the “d=1" case is relevant.), $\left\{x^{\left(i\right)}|i\in \left\{1,\ldots ,n\right\}\right\}$ is the input, and $\left\{z^{\left(i\right)}|i\in \left\{1,\ldots ,n\right\}\right\}$ is the output.*

*One can fit an S2.UncFA model and obtain—via the relationships (5)–(9)—${\hat{\mu }}_{z}$, ${\hat{\Sigma }}_{z}$, $\hat{\Psi }$, $\hat{\Lambda }$, $\hat{\mu }$. Remember that at the test phase (see (4)), the predicted value is*

$${\mathrm{predicted}}_{S2.\mathrm{UncFA}}\left(x^{*}\right)={\hat{\mu }}_{z}+{\hat{\Sigma }}_{z}{\hat{\Lambda }}^{⊤}{\left(\hat{\Lambda }{\hat{\Sigma }}_{z}{\hat{\Lambda }}^{⊤}+\hat{\Psi }\right)}^{-1}\left(x^{*}-\hat{\mu }-\hat{\Lambda }{\hat{\mu }}_{z}\right),\forall x^{*}\in R^{D\times 1}.$$

*One can fit a linear regression model and obtain $\hat{w}$, $\hat{b}$ from (1) and (2). Remember that at the test phase, the predicted value is*

$${\mathrm{predicted}}_{\mathrm{LR}}\left(x^{*}\right)=\hat{w}x^{*}+\hat{b},\forall x^{*}\in R^{D\times 1}.$$

*Then:*

$${\mathrm{predicted}}_{S2.\mathrm{UncFA}}\left(x^{*}\right)={\mathrm{predicted}}_{\mathrm{LR}}\left(x^{*}\right),\forall x^{*}\in R^{D\times 1}.$$

**Proof.**  Let $X=\left[\begin{array}{c} x^{\left(1\right)}\ldots x^{\left(n\right)} \end{array}\right]\in R^{D\times n}$ and $Z=\left[\begin{array}{c} z^{\left(1\right)}\ldots z^{\left(n\right)} \end{array}\right]\in R^{d\times n}$.

We begin by computing $\hat{\Psi }$.

$$\begin{array}{cc} \hat{\Psi } & \overset{\left(9\right)}{=}\frac{1}{n}\sum_{i=1}^{n}\left(x^{\left(i\right)}-\hat{\mu }-\hat{\Lambda }z^{\left(i\right)}\right){\left(x^{\left(i\right)}-\hat{\mu }-\hat{\Lambda }z^{\left(i\right)}\right)}^{⊤}\\ \; & =\frac{1}{n}\sum_{i=1}^{n}(x^{\left(i\right)}{x^{\left(i\right)}}^{⊤}-x^{\left(i\right)}{\hat{\mu }}^{⊤}-x^{\left(i\right)}{z^{\left(i\right)}}^{⊤}{\hat{\Lambda }}^{⊤}-\hat{\mu }{x^{\left(i\right)}}^{⊤}+\hat{\mu }{\hat{\mu }}^{⊤}+\hat{\mu }{z^{\left(i\right)}}^{⊤}{\hat{\Lambda }}^{⊤}-\\ \; & \;-\hat{\Lambda }z^{\left(i\right)}{x^{\left(i\right)}}^{⊤}+\hat{\Lambda }z^{\left(i\right)}{\hat{\mu }}^{⊤}+\hat{\Lambda }z^{\left(i\right)}{z^{\left(i\right)}}^{⊤}{\hat{\Lambda }}^{⊤})\\ \; & =\frac{1}{n}XX^{⊤}-\bar{x}{\hat{\mu }}^{⊤}-\frac{1}{n}XZ^{⊤}{\hat{\Lambda }}^{⊤}-\hat{\mu }{\bar{x}}^{⊤}+\hat{\mu }{\hat{\mu }}^{⊤}+\hat{\mu }{\bar{z}}^{⊤}{\hat{\Lambda }}^{⊤}-\frac{1}{n}\hat{\Lambda }ZX^{⊤}+\\ \; & \;+\hat{\Lambda }\bar{z}{\hat{\mu }}^{⊤}+\frac{1}{n}\hat{\Lambda }ZZ^{⊤}{\hat{\Lambda }}^{⊤}\\ \; & =\frac{1}{n}XX^{⊤}-\frac{1}{n}XZ^{⊤}{\hat{\Lambda }}^{⊤}-\frac{1}{n}\hat{\Lambda }ZX^{⊤}+\frac{1}{n}\hat{\Lambda }ZZ^{⊤}{\hat{\Lambda }}^{⊤}-\bar{x}{\hat{\mu }}^{⊤}-\hat{\mu }{\bar{x}}^{⊤}+\hat{\mu }{\hat{\mu }}^{⊤}+\\ \; & \;+\hat{\Lambda }\bar{z}{\hat{\mu }}^{⊤}+\hat{\mu }{\bar{z}}^{⊤}{\hat{\Lambda }}^{⊤}. \end{array}$$

We substitute $\hat{\mu }$ with $\bar{x}-\hat{\Lambda }\bar{z}$.

$\bar{x}{\hat{\mu }}^{⊤}=\bar{x}{\left(\bar{x}-\hat{\Lambda }\bar{z}\right)}^{⊤}=\bar{x}{\bar{x}}^{⊤}-\bar{x}{\bar{z}}^{⊤}{\hat{\Lambda }}^{⊤}$

$\hat{\mu }{\bar{x}}^{⊤}=\left(\bar{x}-\hat{\Lambda }\bar{z}\right){\bar{x}}^{⊤}=\bar{x}{\bar{x}}^{⊤}-\hat{\Lambda }\bar{z}{\bar{x}}^{⊤}$

$\hat{\mu }{\hat{\mu }}^{⊤}=\left(\bar{x}-\hat{\Lambda }\bar{z}\right){\left(\bar{x}-\hat{\Lambda }\bar{z}\right)}^{⊤}=\bar{x}{\bar{x}}^{⊤}-\bar{x}{\bar{z}}^{⊤}{\hat{\Lambda }}^{⊤}-\hat{\Lambda }\bar{z}{\bar{x}}^{⊤}+\hat{\Lambda }\bar{z}{\bar{z}}^{⊤}{\hat{\Lambda }}^{⊤}$

$\hat{\Lambda }\bar{z}{\hat{\mu }}^{⊤}=\hat{\Lambda }\bar{z}{\left(\bar{x}-\hat{\Lambda }\bar{z}\right)}^{⊤}=\hat{\Lambda }\bar{z}{\bar{x}}^{⊤}-\hat{\Lambda }\bar{z}{\bar{z}}^{⊤}{\hat{\Lambda }}^{⊤}$

$\hat{\mu }{\bar{z}}^{⊤}{\hat{\Lambda }}^{⊤}=\left(\bar{x}-\hat{\Lambda }\bar{z}\right){\bar{z}}^{⊤}{\hat{\Lambda }}^{⊤}=\bar{x}{\bar{z}}^{⊤}{\hat{\Lambda }}^{⊤}-\hat{\Lambda }\bar{z}{\bar{z}}^{⊤}{\hat{\Lambda }}^{⊤}$

We return to compute $\hat{\Psi }$:

(11) Ψ^=1nXX⊤−1nXZ⊤Λ^⊤−1nΛ^ZX⊤+1nΛ^ZZ⊤Λ^⊤−x¯x¯⊤+x¯z¯⊤Λ^⊤−x¯x¯⊤++Λ^z¯z¯⊤+x¯x¯⊤−x¯z¯⊤Λ^⊤−Λ^z¯z¯⊤+Λ^z¯z¯⊤Λ^⊤+Λ^z¯x¯⊤−Λ^z¯z¯⊤Λ^⊤++x¯z¯⊤Λ^⊤−Λ^z¯z¯⊤Λ^⊤=1nXX⊤−1nXZ⊤Λ^⊤−1nΛ^ZX⊤+1nΛ^ZZ⊤Λ^⊤−x¯x¯⊤+Λ^z¯x¯⊤++x¯z¯⊤Λ^⊤−Λ^z¯z¯⊤Λ^⊤.

We continue by computing $\hat{\Lambda }{\hat{\Sigma }}_{z}{\hat{\Lambda }}^{⊤}$.

(12) $$\begin{array}{cc} \; & \hat{\Lambda }{\hat{\Sigma }}_{z}{\hat{\Lambda }}^{⊤}\overset{\left(6\right)}{=}\hat{\Lambda }\left(\frac{1}{n}ZZ^{⊤}-\bar{z}{\bar{z}}^{⊤}\right){\hat{\Lambda }}^{⊤}=\frac{1}{n}\hat{\Lambda }ZZ^{⊤}{\hat{\Lambda }}^{⊤}-\hat{\Lambda }\bar{z}{\bar{z}}^{⊤}{\hat{\Lambda }}^{⊤}. \end{array}$$

We observe that the above term (see (12)) is also included in $\hat{\Psi }$ (see (11)).

(13) $$\begin{array}{cc} {\hat{\Sigma }}_{z}{\hat{\Lambda }}^{⊤} & \overset{\left(7\right)}{=}\left(\frac{1}{n}ZZ^{⊤}-\bar{z}{\bar{z}}^{⊤}\right){\left(n\bar{z}{\bar{z}}^{⊤}-ZZ^{⊤}\right)}^{-1}\left(n\bar{z}{\bar{x}}^{⊤}-ZX^{⊤}\right)\\ \; & =\left(\frac{1}{n}ZZ^{⊤}-\bar{z}{\bar{z}}^{⊤}\right)\frac{-1}{n}{\left(\frac{1}{n}ZZ^{⊤}-\bar{z}{\bar{z}}^{⊤}\right)}^{-1}\left(n\bar{z}{\bar{x}}^{⊤}-ZX^{⊤}\right)\\ \; & =\frac{1}{n}ZX^{⊤}-\bar{z}{\bar{x}}^{⊤}. \end{array}$$

Since ${\left(\hat{\Lambda }{\hat{\Sigma }}_{z}{\hat{\Lambda }}^{⊤}\right)}^{⊤}=\hat{\Lambda }{\hat{\Sigma }}_{z}^{⊤}{\hat{\Lambda }}^{⊤}=\hat{\Lambda }{\hat{\Sigma }}_{z}{\hat{\Lambda }}^{⊤}\Rightarrow \hat{\Lambda }{\hat{\Sigma }}_{z}{\hat{\Lambda }}^{⊤}$ is symmetric.

We have that:

(14) $$\begin{array}{c} \hat{\Lambda }{\hat{\Sigma }}_{z}{\hat{\Lambda }}^{⊤}=\hat{\Lambda }\left({\hat{\Sigma }}_{z}{\hat{\Lambda }}^{⊤}\right)\overset{\left(13\right)}{=}\hat{\Lambda }\left(\frac{1}{n}ZX^{⊤}-\bar{z}{\bar{x}}^{⊤}\right)=\frac{1}{n}\hat{\Lambda }ZX^{⊤}-\hat{\Lambda }\bar{z}{\bar{x}}^{⊤}. \end{array}$$

We also have that:

(15) $$\begin{array}{cc} \hat{\Lambda }{\hat{\Sigma }}_{z}{\hat{\Lambda }}^{⊤} & ={\left(\hat{\Lambda }{\hat{\Sigma }}_{z}{\hat{\Lambda }}^{⊤}\right)}^{⊤}\overset{\left(13\right)}{=}{\left(\frac{1}{n}\hat{\Lambda }ZX^{⊤}-\hat{\Lambda }\bar{z}{\bar{x}}^{⊤}\right)}^{⊤}=\frac{1}{n}XZ^{⊤}{\hat{\Lambda }}^{⊤}-\bar{x}{\bar{z}}^{⊤}{\hat{\Lambda }}^{⊤}. \end{array}$$

We continue by computing $\hat{\Lambda }{\hat{\Sigma }}_{z}{\hat{\Lambda }}^{⊤}+\hat{\Psi }$.

As we have already noticed above, $\hat{\Lambda }{\hat{\Sigma }}_{z}{\hat{\Lambda }}^{⊤}$ is also included in $\hat{\Psi }$ (see (11) and (12)). In the computation of $\hat{\Lambda }{\hat{\Sigma }}_{z}{\hat{\Lambda }}^{⊤}+\hat{\Psi }$, we replace $\hat{\Lambda }{\hat{\Sigma }}_{z}{\hat{\Lambda }}^{⊤}$ once with (14) and then with (15). We get:

(16) Λ^Σ^zΛ^⊤+Ψ^=(14)(15)(11)(12)1nXX⊤−1nXZ⊤Λ^⊤−1nΛ^ZX⊤−x¯x¯⊤+Λ^z¯x¯⊤+        +x¯z¯⊤Λ^⊤+1nΛ^ZX⊤−Λ^z¯x¯⊤+1nXZ⊤Λ^⊤−x¯z¯⊤Λ^⊤=1nXX⊤−x¯x¯⊤.

Observation: The result is exactly the maximum likelihood estimate of the covariance matrix Σ of the input data set if x∼N(μ,Σ): $\Sigma_{\mathrm{MLE}}=\frac{1}{n}XX^{⊤}-\bar{x}{\bar{x}}^{⊤}$. This is natural because according to the relationship (4) we have $x∼N(\mu ,\Lambda \Sigma_{z}\Lambda^{⊤}+\Psi )$, and there are enough free parameters in $\Lambda \Sigma_{z}\Lambda^{⊤}+\Psi$, i.e., $dD+\frac{d^{2}-d}{2}+d+\frac{D^{2}-D}{2}+D$ free parameters—dD in Λ, $\frac{d^{2}-d}{2}$ in $\Sigma_{z}$, $\frac{D^{2}-D}{2}$ in Ψ, for it to become $\Sigma_{\mathrm{MLE}}=\frac{1}{n}XX^{⊤}-\bar{x}{\bar{x}}^{⊤}$, since Σ has $\frac{D^{2}-D}{2}+D$ free parameters.

We return to the initial computation:

${\mathrm{predicted}}_{S2.\mathrm{UncFA}}\left(x^{*}\right)=$

$\overset{\left(4\right)}{=}{\hat{\mu }}_{z}+{\hat{\Sigma }}_{z}{\hat{\Lambda }}^{⊤}{\left(\hat{\Lambda }{\hat{\Sigma }}_{z}{\hat{\Lambda }}^{⊤}+\hat{\Psi }\right)}^{-1}\left(x^{*}-\hat{\mu }-\hat{\Lambda }{\hat{\mu }}_{z}\right)$

=(5)(13)(16)z¯+1nZX⊤−z¯x¯⊤1nXX⊤−x¯x¯⊤−1(x*−x¯+Λ^z¯−Λ^z¯)

$=\left(\frac{1}{n}ZX^{⊤}-\bar{z}{\bar{x}}^{⊤}\right){\left(\frac{1}{n}XX^{⊤}-\bar{x}{\bar{x}}^{⊤}\right)}^{-1}x^{*}+\bar{z}-\left(\frac{1}{n}ZX^{⊤}-\bar{z}{\bar{x}}^{⊤}\right){\left(\frac{1}{n}XX^{⊤}-\bar{x}{\bar{x}}^{⊤}\right)}^{-1}\bar{x}$

$=\left(n\bar{z}{\bar{x}}^{⊤}-ZX^{⊤}\right){\left(n\bar{x}{\bar{x}}^{⊤}-XX^{⊤}\right)}^{-1}x^{*}+\bar{z}-\left(n\bar{z}{\bar{x}}^{⊤}-ZX^{⊤}\right){\left(n\bar{x}{\bar{x}}^{⊤}-XX^{⊤}\right)}^{-1}\bar{x}$

$\overset{\left(1\right)}{=}\hat{w}x^{*}+\bar{z}-\hat{w}\bar{x}$

$\overset{\left(2\right)}{=}\hat{w}x^{*}+\hat{b}$

$={\mathrm{predicted}}_{\mathrm{LR}}\left(x^{*}\right),\forall x^{*}\in R^{D\times 1}.$ □

#### 4.1.3. The S2.FA Model. A New Approach for LR When D>>n

Since FA can be used to estimate the density of a data set when D>>n, and S2.UncFA is equivalent to LR, we consider S2.FA as a new approach to extend LR when D>>n besides the two solutions mentioned in Section 2.

### 4.2. The S3.FA Model

Factor analysis is a classic generative unsupervised model. Its supervised counterpart is S2.FA as shown in the previous subsection. Those two can be merged into a semisupervised model that we propose here, named simple-semisupervised factor analysis:

$z∼N(\mu_{z},\sigma_{z}^{2})$-either observed or latent variable, z∈R, $\mu_{z}\in R$, $\sigma_{z}\in R_{+}^{*}$

x|z∼N(μ+Λz,Ψ), $x\in R^{D\times 1},\mu \in R^{D\times 1},\Lambda \in R^{D\times 1}$, and $\Psi \in R^{D\times D}$ a diagonal matrix.

If we were to speak about Gaussian naive Bayes and the GMM, good hints for combining these two supervised–unsupervised counterparts into a semisupervised model can be found in [12]. We applied those hints for our supervised–unsupervised counterparts—S2.FA and FA—and created an EM algorithm to fit an S3.FA model. For the step-by-step algorithm and the matrix form of the algorithm, see Appendix C. For more elaborate notations and the proof, see [8] pp. 26–34.

### 4.3. The MS3.FA Model

The algorithm that fits an S3.FA model can be adapted also for the case when not all the components of *x* are known. We call the resulted model missing simple-semisupervised factor analysis:

$z∼N(\mu_{z},\sigma_{z}^{2})$-either observed or latent variable, z∈R, $\mu_{z}\in R$, $\sigma_{z}\in R_{+}^{*}$

x|z∼N(μ+Λz,Ψ), $x\in R^{D\times 1},\mu \in R^{D\times 1},\Lambda \in R^{D\times 1}$, and $\Psi \in R^{D\times D}$ a diagonal matrix; each component of *x*: $x_{1},\ldots ,x_{D}$ is either observed or latent.

The resulting algorithm that fits a MS3.FA model is an EM algorithm. For the step-by-step algorithm, see Appendix D. For more elaborate notations and the proof, see [8] pp. 34–37.

## 5. Experiments

In this section, we include the experiments we carried out on data with D>>n using the S2.FA, S3.FA, and MS3.FA models, comparing them with other methods. In all the experiments, we computed errors between the real values and the predicted values; the metric we used is mean squared error (MSE):

$$\mathrm{MSE}=\frac{1}{N}\sum_{i=1}^{N}{\left({\mathrm{real}}_{i}-{\mathrm{predicted}}_{i}\right)}^{2},$$

where *N* is the number of the unknown elements whose real and predicted values are ${\mathrm{real}}_{i}$ and ${\mathrm{predicted}}_{i}$, respectively; an unknown element represents an output number for S2.FA and S3.FA or an input/output number for MS3.FA. We ran each experiment five times and computed a 95% confidence interval using the *t*-distribution. Furthermore, in each experiment we used the same three data sets:

- Gas sensor array under flow modulation data set (http://archive.ics.uci.edu/ml/datasets/Gas+sensor+array+under+flow+modulation; accessed on 31 July 2021) [13]: 58 observations, 432 input attributes;
- atp1d—the airline ticket price; 1D refers to the fact that the target price is in the next day—(https://www.openml.org/d/41475; accessed on 31 July 2021) [14]: 337 observations, 411 input attributes; 370 after preprocessing: see below;
- m5spec—corn measured on a NIR spectrometer: mp5 instrument—(http://www.eigenvector.com/data/Corn; accessed on 31 July 2021): 80 observations, 700 input attributes.

All three of these data sets have multiple outputs, but for each data set, we selected only the first output column that appears in the text data file and used it as the output: *ace_conc* for gas sensor array under flow modulation data set, *LBL_ALLminpA_fut_001* for atp1d, the first column in the *propvals* file for m5spec.

We preprocessed each data set simply by dropping the constant columns. Only the second data set has constant columns: from 432 columns, we obtain 370.

### 5.1. The S2.FA Model: Experiment

The experiment concerning S2.FA covers the comparison of the three solutions presented so far for LR when D>>n:

- Moore–Penrose inverse
- ridge regression—L2 regularization
- S2.FA.

Each data set was split into a training part—80%—and a testing part—20%. If the model had hyperparameters, ridge regression, the training part was also split into a new training part—60% of the whole data set—and a validation part—20% of the whole data set—in order to be able to set the hyperparameters (for ridge regression, we used a simple technique: pick $\alpha \in \{10^{2},10^{1.9},10^{1.8},\ldots ,10^{-1.9},10^{-2}\}$, which attains the minimum validation error); after setting the hyperparameters, we train a new model on the initial training part—80% of the whole data set—and obtain the final model. All of the MSE errors are reported on the testing part and shown in Table 1.

As one may notice, the best method is different for each data set, so our general advice is to use all the methods on a given data set and pick the best one.

### 5.2. The S3.FA Model: Experiment

The experiment concerning S3.FA includes an analysis of algorithms for semisupervised regression when D>>n:

- Moore–Penrose inverse—a supervised method
- S2.FA—a supervised method
- S3.FA—a semisupervised method
- label propagation [15]—a semisupervised method: we used the function
  sslLabelProp in the SSL R package [16] with the parameter alpha set to 1.

**Figure 1:**
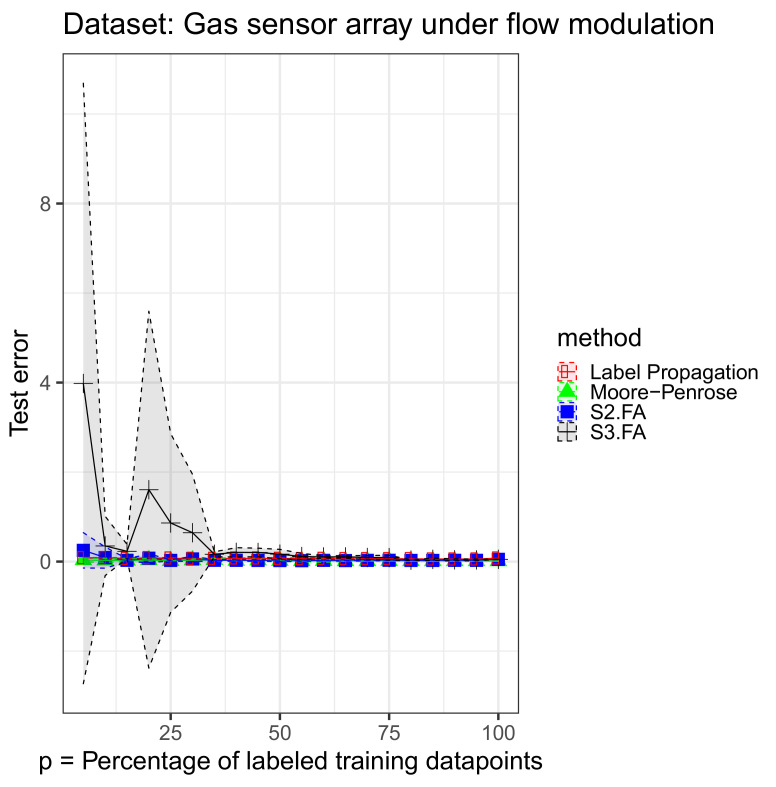
Simple-semisupervised factor analysis (S3.FA) experiment: MSE 95% confidence intervals on the gas sensor array under flow modulation data set using four methods for semisupervised regression when D>>n; p∈{5%,10%,15%,…,100%} of the training output labels are retained.

**Figure 2:**
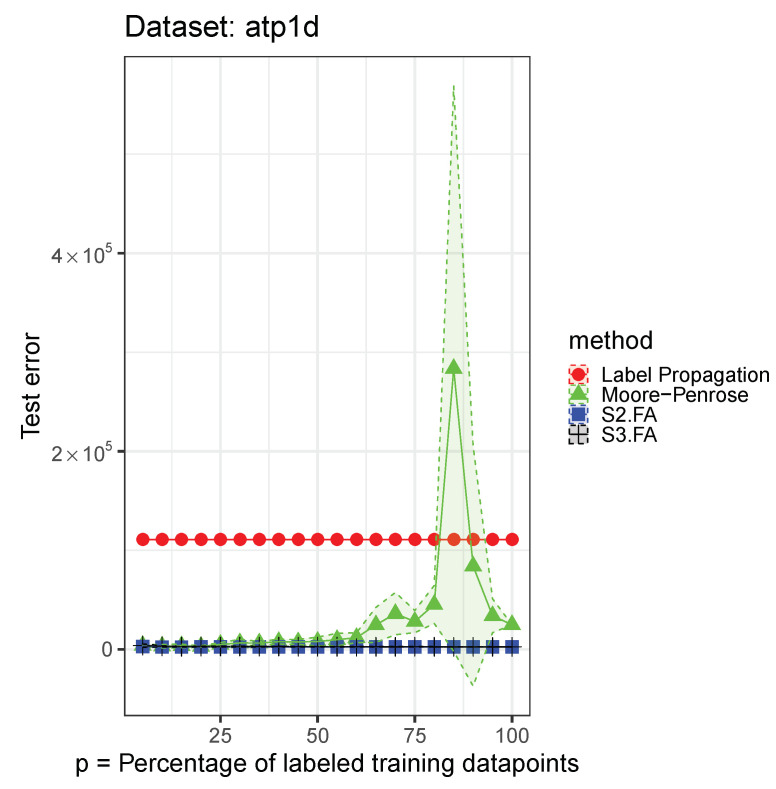
S3.FA experiment: MSE 95% confidence intervals on the *atp1d* data set using four methods for semisupervised regression when D>>n; p∈{5%,10%,15%,…,100%} of the training output labels are retained.

**Figure 3:**
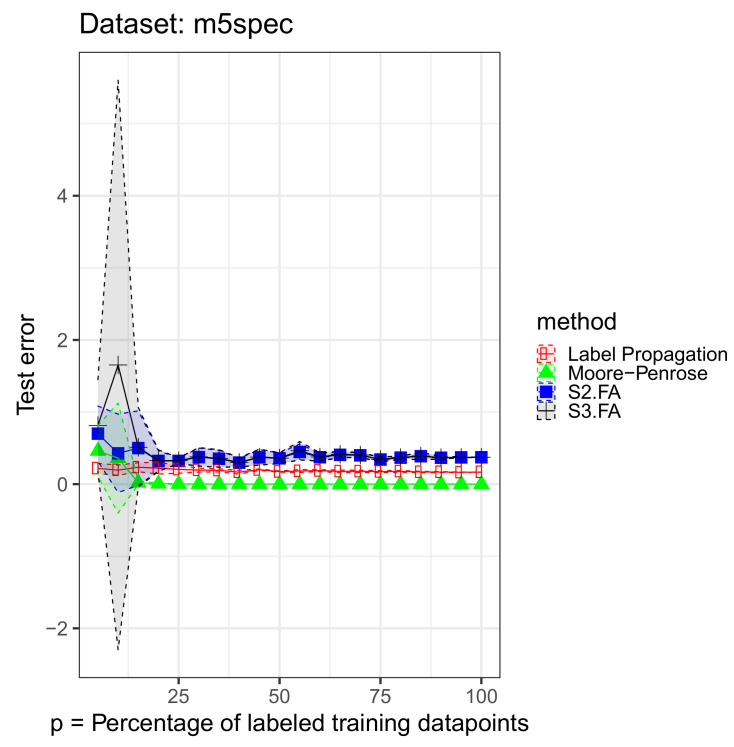
S3.FA experiment: MSE 95% confidence intervals on the *m5spec* data set using four methods for semisupervised regression when D>>n; p∈{5%,10%,15%,…,100%} of the training output labels are retained.

Each data set was split into a training part—80%—and a testing part—20%. We retained from the training part 5%, 10%, 15%, 20%, 25%, …, 100% of the output labels. For the supervised methods, we used only the labeled data in the training set, and for the semisupervised methods, we used the full training set when fitting the model. We initialized the S3.FA method with the fitted parameters returned by the S2.FA algorithm. All of the MSE errors are reported on the testing part and shown in Figure 1, Figure 2 and Figure 3—inspired from [17]—and Table 2. The figures contain all the results from using 5% to 100% of the output labels, but we include less information in the table for brevity.

We notice that on the selected data sets, S3.FA returns poorer results even than S2.FA, which uses only the labeled data. As in the previous experiment, the best method is also data-dependent. The models that show a greater amount of variability compared to the others are S3.FA—in two data sets—and Moore–Penrose—in one data set. Moreover, as expected, the errors tend to decrease as the percentage of labeled training data points increases; the plots do not help us in this regard, but this decrease can be seen numerically in Table 2.

### 5.3. The MS3.FA Model: Experiment

The experiment concerning MS3.FA includes a comparison of two different types of algorithms for data imputation when D>>n:

- Mean imputation: for a given attribute (input column), compute its mean ignoring the missing values, then replace the missing data on that attribute with this computed mean
- MS3.FA.

We also tried two other R packages: mice [18] and Amelia [19], but they could not be applied successfully on our data sets perhaps because they have a peculiarity: D>>n.

**Figure 4:**
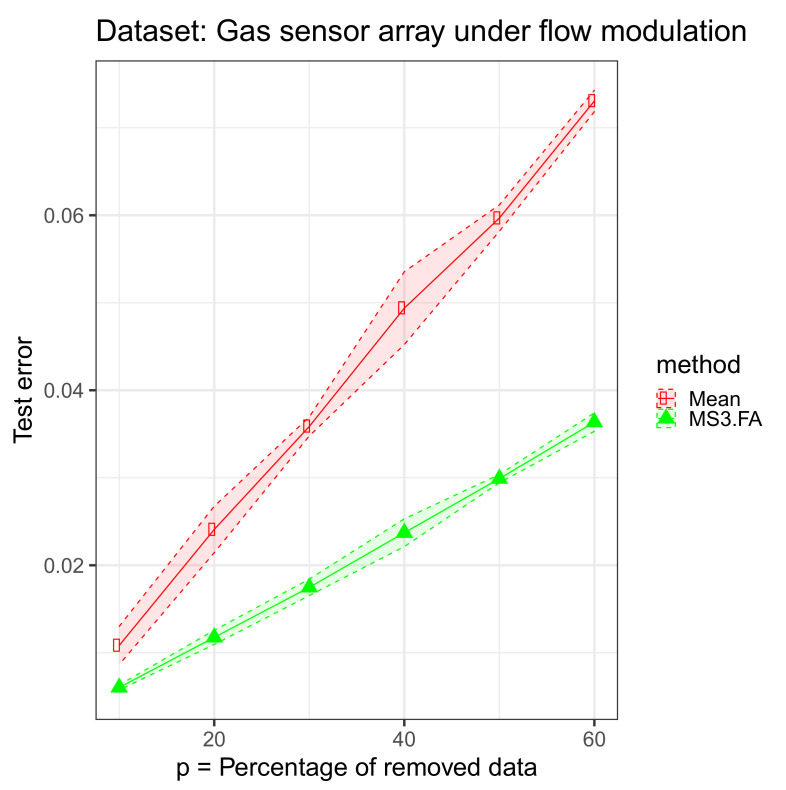
Missing simple-semisupervised factor analysis (MS3.FA) experiment: MSE 95% confidence intervals on the gas sensor array under flow modulation data set using two methods for imputing missing data when D>>n; p∈{10%,20%,30%,40%,50%,60%} of the input data are removed.

**Figure 5:**
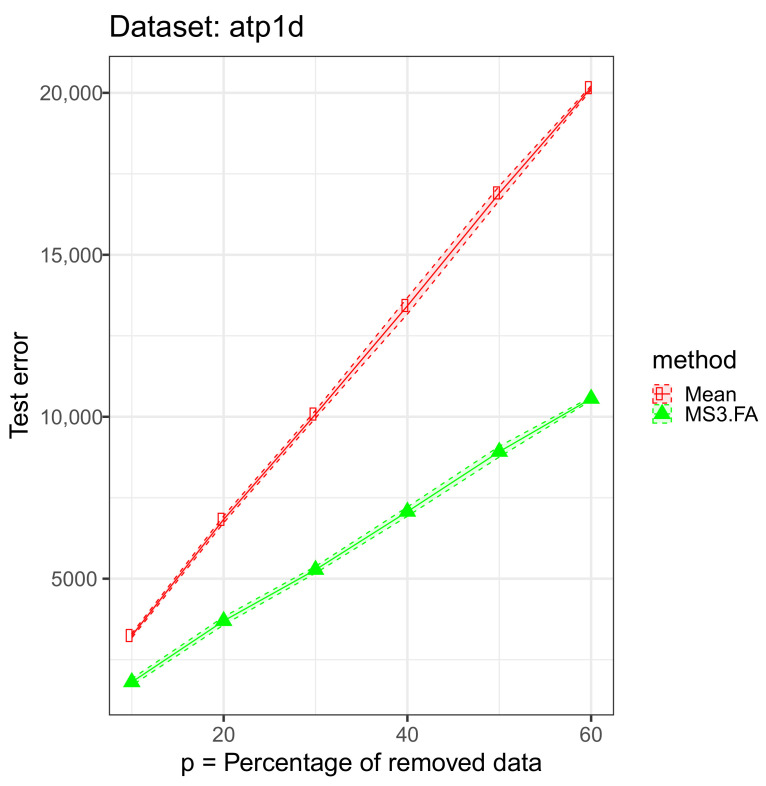
MS3.FA experiment: MSE 95% confidence intervals on the *atp1d* data set using two methods for imputing missing data when D>>n; p∈{10%,20%,30%,40%,50%,60%} of the input data are removed.

**Figure 6:**
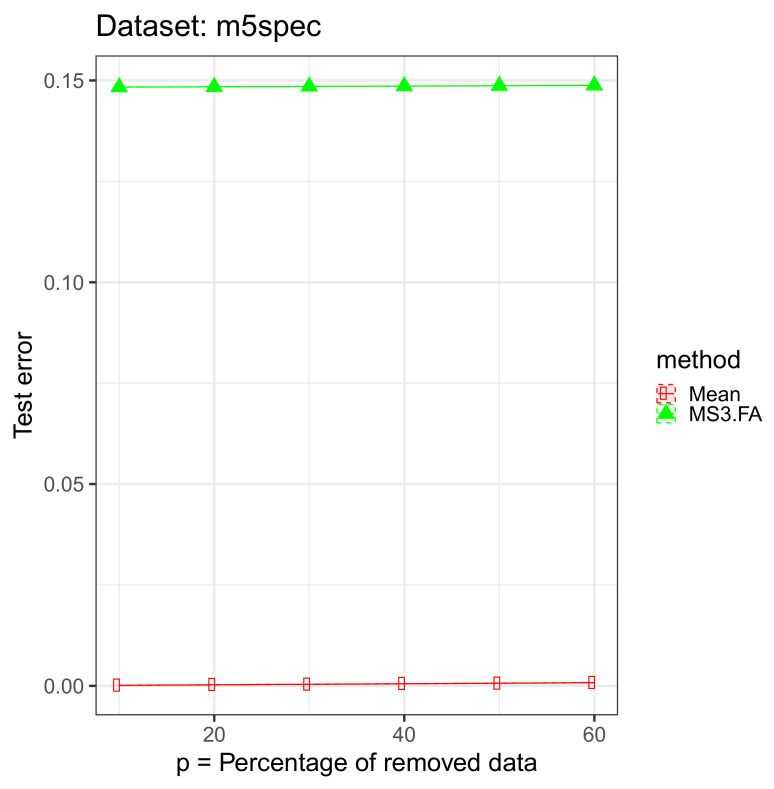
MS3.FA experiment: MSE 95% confidence intervals on the *m5spec* data set using two methods for imputing missing data when D>>n; p∈{10%,20%,30%,40%,50%,60%} of the input data are removed.

For each data set, we removed 10%, 20%, 30%, 40%, 50%, and 60% of the input (We could have added missing data also in the output, but we wanted to focus on the missing input data scenario and not on the semisupervised case.) cells and imputed those via the above mentioned algorithms. The results are presented in Figure 4, Figure 5 and Figure 6 and in Table 3.

From these results, we discover that MS3.FA is better than mean imputation on two data sets, and, as expected, the error increases as the percentage of missing data increases.

## 6. Conclusions and Future Work

The initial purpose of this paper was to extend an already existing model: factor analysis. We developed its supervised counterpart (S2.FA) and noticed that the unconstrained version (S2.UncFA) is equivalent to linear regression. Because FA is applied in density estimation when the dimensionality of the data is greater than the number of samples, and because of the already mentioned equivalence, the purpose of the paper became to analyze this new method of applying LR when D>>n, i.e., via S2.FA. Since FA and S2.FA are generative models and are unsupervised–supervised counterparts, we combined both into a new model S3.FA as an extension of LR to semisupervised learning when D>>n. The final extension regards missing data; it is called MS3.FA. We developed an R package (s2fa) with these algorithms; it can be found on GitHub. The experimental parts included several comparisons in the D>>n scenario:

- of S2.FA with other techniques extending LR to the D>>n case,
- of S3.FA with other (semi)supervised regression methods,
- of MS3.FA with another data imputation algorithm.

The bottom line is that we do not necessarily recommend S3.FA for semisupervised regression since our results suggest that it gives poor results, but we encourage the consideration of S2.FA for regression and MS3.FA for missing data imputation as algorithms to be compared with others on a given data set.

As for future work, we could further explore the S2.FA, S3.FA, and MS3.FA algorithms when *z* is a real vector, not just a real number. Moreover, we can experiment with the PPCA version of the algorithms. Questions regarding the time complexity—empirical or not—can be also addressed; we expect the fitting time to be impractical if the number of columns is large. Because there are models such as mixture of factor analyzers [20] and mixture of linear regression models ([21] Section 14.5.1), another research direction involves mixtures of S2.FAs. Another idea would be to investigate the memory resources required by the algorithms when the data set increases and also to consider scalable systems such as Spark [22] for implementation.

## Figures and Tables

**Table 1:** Simple-supervised factor analysis (S2.FA) experiment: mean squared error (MSE) 95% confidence intervals on three data sets using three methods for regression when D>>n; the best MSE means are marked in bold.

Data Set/Method	Moore–Penrose	Ridge Regression	S2.FA
**Gas sensor array under flow modulation**	0.0251 ± 0.0254	**0.0062** ± 0.007	0.0452 ± 0.0208
**atp1d**	94,627.7239 ± 80,183.0076	27,770.9253 ± 42,887.5216	**4724.2957** ± 1616.3341
**m5spec**	**0.00004** ± 0.00001	0.02676 ± 0.01372	0.37344 ± 0.25025

**Table 2:** S3.FA experiment: MSE 95% confidence intervals on three data sets using four methods for semisupervised regression when D>>n; p∈{5%,10%,15%,30%,50%,70%} of the training output labels are retained; the best MSE means are marked in bold.

Data Set	Method	p=5%	p=10%	p=15%
**Gas sensor** **array under** **flow modulation**	Moore–Penrose	**0.034** ± 0.0437	**0.0292** ± 0.0421	**0.0267** ± 0.0326
	S2.FA	0.2511 ± 0.3953	0.0899 ± 0.2367	0.0285 ± 0.0333
	S3.FA	3.9799 ± 6.7087	0.349 ± 0.6626	0.2294 ± 0.1561
	Label Propagation	0.0853 ± 0.0073	0.0825 ± 0.0051	0.0708 ± 0.013
**atp1d**	Moore–Penrose	3763.2939 ± 749.0966	3079.5042 ± 1842.7676	3428.7567 ± 1722.6437
	S2.FA	**2706.9906** ± 477.3245	**2339.3504** ± 324.8581	**2279.0802** ± 99.5443
	S3.FA	3771.605 ± 1563.6543	2972.1137 ± 639.6724	2633.0816 ± 274.7409
	Label Propagation	110,820.3235 ± 0	110,820.3235 ± 0	110,820.3235 ± 0
**m5spec**	Moore–Penrose	0.4602 ± 0.354	0.3609 ± 0.7631	**0.0187** ± 0.0221
	S2.FA	0.7003 ± 0.3834	0.4269 ± 0.5441	0.4999 ± 0.5172
	S3.FA	0.8133 ± 0.6366	1.653 ± 3.9497	0.5118 ± 0.5503
	Label Propagation	**0.2175** ± 0.0707	**0.1939** ± 0.0706	0.2343 ± 0.0624
**Data Set**	**Method**	p=30%	p=50%	p=70%
**Gas sensor** **array under** **flow modulation**	Moore–Penrose	**0.0313** ± 0.0259	**0.0192** ± 0.0145	**0.0132** ± 0.0075
	S2.FA	0.0588 ± 0.0576	0.0233 ± 0.0224	0.0279 ± 0.0116
	S3.FA	0.645 ± 1.3044	0.1666 ± 0.1059	0.0912 ± 0.0443
	Label Propagation	0.0668 ± 0.0086	0.0675 ± 0.018	0.0605 ± 0.0122
**atp1d**	Moore–Penrose	6401.7587 ± 3602.9182	7907.484 ± 4449.2312	36,041.874 ± 21,300.704
	S2.FA	**2444.9001** ± 404.2393	**2439.162** ± 108.3605	**2399.7948** ± 160.1038
	S3.FA	2760.6602 ± 391.9217	2659.6472 ± 112.9795	2521.9861 ± 222.3765
	Label Propagation	110,820.3235 ± 0	110,820.3235 ± 0	110,820.3235 ± 0
**m5spec**	Moore–Penrose	**0.00057** ± 0.00085	**0.00009** ± 0.00008	**0.00009** ± 0.00004
	S2.FA	0.37449 ± 0.12508	0.35796 ± 0.07275	0.3995 ± 0.03164
	S3.FA	0.37865 ± 0.12808	0.36181 ± 0.07463	0.40419 ± 0.03415
	Label Propagation	0.19391 ± 0.02754	0.17424 ± 0.00857	0.17752 ± 0.01545

**Table 3:** MS3.FA experiment: MSE 95% confidence intervals on three data sets using two methods for imputing missing data when D>>n; p∈{10%,20%,30%,40%,50%,60%} of the input data are removed; the best MSE means are marked in bold.

Data Set	Method	p=10%	p=20%	p=30%
**Gas sensor array under** **flow modulation**	Mean	0.0108 ± 0.0022	0.0241 ± 0.0027	0.0359 ± 0.0011
	MS3.FA	**0.00603** ± 0.00029	**0.01176** ± 0.00081	**0.01747** ± 0.00094
**atp1d**	Mean	3239.9757 ± 68.2511	6831.0884 ± 105.6119	10,077.1798 ± 117.4693
	MS3.FA	**1807.7748** ± 105.03	**3697.1146** ± 124.2197	**5276.899** ± 104.6252
**m5spec**	Mean	**0.00013** ± 0.00001	**0.00026** ± 0	**0.00039** ± 0.00001
	MS3.FA	0.14835 ± 0.000002	0.148433 ± 0.000002	0.148518 ± 0.000002
**Data Set**	**Method**	p=40%	p=50%	p=60%
**Gas sensor array under** **flow modulation**	Mean	0.0494 ± 0.0042	0.0597 ± 0.0015	0.0731 ± 0.0012
	MS3.FA	**0.02372** ± 0.00159	**0.0299** ± 0.00047	**0.0364** ± 0.0011
**atp1d**	Mean	13,423.3625 ± 252.7735	16,899.3166 ± 223.6971	20,156.2493 ± 62.1299
	MS3.FA	**7071.6793** ± 143.3232	**8921.5319** ± 165.5759	**10,558.5723** ± 53.1182
**m5spec**	Mean	**0.000521** ± 0.000005	**0.000658** ± 0.000008	**0.00080** ± 0.000005
	MS3.FA	0.1486 ± 0.00001	0.14869 ± 0.00001	0.148781 ± 0.000001

## Data Availability

Publicly available data sets were analyzed in this study. This data can be found here: http://archive.ics.uci.edu/ml/datasets/Gas+sensor+array+under+flow+modulation (accessed on 31 July 2021) for the gas sensor array under flow modulation data set, https://www.openml.org/d/41475 (accessed on 31 July 2021) for atp1d, http://www.eigenvector.com/data/Corn (accessed on 31 July 2021) for m5spec.

## Abbreviations

   The following abbreviations are used in this manuscript:

GMM	Gaussian mixture model
LR	Linear regression
EM	Expectation–maximization
KL	Kullback–Leibler
FA	Factor analysis
PCA	Principal component analysis
PPCA	Probabilistic principal component analysis
GPLVM	Gaussian process latent variable model
MLE	Maximum likelihood estimation
UncFA	Unconstrained factor analysis
S2.UncFA	Simple-supervised unconstrained factor analysis
S2.FA	Simple-supervised factor analysis
S2.PPCA	Simple-supervised probabilistic principal component analysis
S3.UncFA	Simple-semisupervised unconstrained factor analysis
S3.FA	Simple-semisupervised factor analysis
S3.PPCA	Simple-semisupervised probabilistic principal component analysis
MS3.UncFA	Missing simple-semisupervised unconstrained factor analysis
MS3.FA	Missing simple-semisupervised factor analysis
MS3.PPCA	Missing simple-semisupervised probabilistic principal component analysis
MSE	Mean squared error
ELBO	Evidence lower bound

## Appendix A. On the Expectation–Maximization Algorithm

This section provides theoretical details on the EM algorithm [23]. These are relevant in order to establish a link between EM and the information theory field.

A latent variable model assumes that the data we observed—usually, denoted by the random variable *X*—is not complete: there is also some latent data modeled via random variables—usually, denoted by *Z*. Often, pairs consisting of an observed point and a latent one constitute the complete dataset, e.g., in a GMM the latent data is the cluster number and such a number is assigned to each point in the observed dataset.

Usually, in a latent variable model the likelihood of the observed data is not tractable—although there are exceptions, like in GMM or factor analysis—and therefore we cannot maximize it directly. Instead we maximize a lower bound for the log-likelihood function called ELBO (evidence lower bound). To simplify the discussion, we consider that we have one single observed datapoint, *x*. We will also consider the case where *Z* is continuous; when *Z* is discrete the ∫ sign is replaced by the ∑ sign.

Let *q* be any distribution over *z*, where the *z* values are the possible values of the random variable *Z*. The log-likelihood of the observed datapoint *x* is:   

$$\begin{array}{cc} \log p\left(x;\theta \right) & \overset{\mathrm{marginal}}{=}\log\int_{z}p\left(x,z;\theta \right)dz\\ & =\log\int_{z}\frac{q(z)}{q(z)}p\left(x,z;\theta \right)dz\\ & =\log\int_{z}q\left(z\right)\frac{p(x,z;\theta )}{q(z)}dz\\ & =\log E_{q}\left[\frac{p(x,z;\theta )}{q(z)}\right]\\ & \overset{\mathrm{Jensen}}{\geq }\underset{\mathrm{ELBO}(\theta ,q)}{\underset{︸}{E_{q}\left[\log\frac{p(x,z;\theta )}{q(z)}\right]}}. \end{array}$$

Furthermore, the following relationships important for the E step of the EM algorithm—see below—can be proven:

$$\begin{array}{cc} \log p(x;\theta )-\mathrm{ELBO}(\theta ,q) & =\mathrm{KL}(q(\cdot )||p(\cdot |x;\theta ))\\ \; & ⇕\\ \log p(x;\theta ) & =\mathrm{ELBO}(\theta ,q)+\mathrm{KL}(q(\cdot )||p(\cdot |x;\theta ))\\ \; & ⇕\\ \mathrm{ELBO}(\theta ,q) & =\log p(x;\theta )-\mathrm{KL}(q(\cdot )||p(\cdot |x;\theta )). \end{array}$$

Moreover, the following relationships important for the M step of the EM algorithm—see below—can be proven:

$$\begin{array}{cc} \mathrm{ELBO}(\theta ,q) & \overset{\mathrm{def}.}{=}E_{q}\left[\log\frac{p(x,z;\theta )}{q(z)}\right]\\ \; & =\int_{z}q\left(z\right)\log\frac{p(x,z;\theta )}{q(z)}dz\\ \; & =\int_{z}q\left(z\right)\log p(x,z;\theta )dz-\int_{z}q\left(z\right)\log q(z)dz\\ \; & =E_{q}\left[\log p\left(x,z;\theta \right)\right]+H\left(q\right). \end{array}$$

Now, instead of carrying out $\max_{\theta }\log p\left(x;\theta \right)$ we will execute $\max_{\theta ,q}\mathrm{ELBO}\left(\theta ,q\right)$, since ELBO is a lower bound for the log-likelihood of *x* and hence its maximization will not hurt the process of maximizing logp(x;θ).

The ELBO can be maximized in at least two ways:

- via (block) coordinate ascent
  The resulting meta-algorithm is the EM algorithm.
  In fact this is the case for many classic models—EM for GMM [3], EM for factor analysis [5] etc.—.
  EM is an iterative algorithm and an iteration encompasses two steps:
  1. E step:
    $q^{\left(t\right)}=\arg\max_{q}\mathrm{ELBO}\left(\theta^{\left(t-1\right)},q\right)$ for $\theta^{\left(t-1\right)}$ fixed—from the previous iteration.
    Since ELBO(θ,q)=logp(x;θ)−KL(q(·)||p(·|x;θ)), we have:   
    $$\begin{array}{cc} q^{\left(t\right)} & =\arg\max_{q}\mathrm{ELBO}\left(\theta^{\left(t-1\right)},q\right)\\ \; & =\arg\max_{q}(\log p\left(x;\theta^{\left(t-1\right)}\right)-\mathrm{KL}(q\left(\cdot \right)||p\left(\cdot |x;\theta^{\left(t-1\right)}\right)))\\ \; & =\arg\min_{q}\mathrm{KL}(q\left(\cdot \right)||p\left(\cdot |x;\theta^{\left(t-1\right)}\right))\overset{\mathrm{KL}\;\mathrm{property}}{=}p\left(\cdot |x;\theta^{\left(t-1\right)}\right). \end{array}$$
    (In this case, we have $\log p\left(x;\theta^{\left(t-1\right)}\right)=\mathrm{ELBO}\left(\theta^{\left(t-1\right)};q^{\left(t\right)}\right)$.)
    So, we obtained the distribution $q^{\left(t\right)}$ as a posterior distribution. Although in classic models where conjugate priors are used it is tractable to compute $p(\cdot |x;\theta^{\left(t-1\right)})$—this type of inference is called analytical inference—, in other models this is not the case and a solution to this shortcoming is represented by approximate/variational inference.
  2. M step:
    $\theta^{\left(t\right)}=\arg\max_{\theta }\mathrm{ELBO}\left(\theta ,q^{\left(t\right)}\right)$ for $q^{\left(t\right)}$ fixed—from the E step.
    Since $\mathrm{ELBO}\left(\theta ,q\right)=E_{q}\left[\log p\left(x,z;\theta \right)\right]+H\left(q\right)$, we have:
    $$\begin{array}{cc} \theta^{\left(t\right)} & =\arg\max_{\theta }\mathrm{ELBO}\left(\theta ,q^{\left(t\right)}\right)\\ \; & =\arg\max_{\theta }\left(E_{q^{\left(t\right)}}\left[\log p\left(x,z;\theta \right)\right]+H\left(q^{\left(t\right)}\right)\right)\\ \; & =\arg\max_{\theta }E_{q^{\left(t\right)}}\left[\log p\left(x,z;\theta \right)\right]. \end{array}$$
    So, we obtained a relatively simpler term to maximize. Note that the maximization is further customized using the probabilistic assumptions at hand.
- via gradient ascent: this is the case of Variational Autoencoder [24] which will not be discussed since it is not necessarily relevant to this study.

## Appendix B. S2.FA

**Algorithm A1** S2.FA—nonmatrix form.
1: **function**Train( $\left\{\left(x^{\left(i\right)},z^{\left(i\right)}\right)|i\in \left\{1,\ldots ,n\right\}\right\}$) 2: $\bar{x}=\frac{\sum_{i=1}^{n}x^{\left(i\right)}}{n}$ 3: ${\hat{\mu }}_{z}=\frac{\sum_{i=1}^{n}z^{\left(i\right)}}{n}=\bar{z}$ 4: ${\hat{\Sigma }}_{z}=\frac{\sum_{i=1}^{n}\left(z^{\left(i\right)}-{\hat{\mu }}_{z}\right){\left(z^{\left(i\right)}-{\hat{\mu }}_{z}\right)}^{⊤}}{n}$ 5: $\hat{\Lambda }=\left(n\bar{x}{\bar{z}}^{⊤}-\sum_{i=1}^{n}x^{\left(i\right)}{z^{\left(i\right)}}^{⊤}\right){\left(n\bar{z}{\bar{z}}^{⊤}-\sum_{i=1}^{n}z^{\left(i\right)}{z^{\left(i\right)}}^{⊤}\right)}^{-1}$ 6: $\hat{\mu }=\bar{x}-\hat{\Lambda }\bar{z}$ 7: $\hat{\Psi }=\mathrm{diag}\left(\frac{\sum_{i=1}^{n}\left(x^{\left(i\right)}-\hat{\mu }-\Lambda z^{\left(i\right)}\right){\left(x^{\left(i\right)}-\hat{\mu }-\Lambda z^{\left(i\right)}\right)}^{⊤}}{n}\right)$ 8: **return** ( ${\hat{\mu }}_{z}$, ${\hat{\Sigma }}_{z}$, $\hat{\Lambda }$, $\hat{\mu }$, $\hat{\Psi }$) 9: **function**Test( $x^{*}$,( ${\hat{\mu }}_{z}$, ${\hat{\Sigma }}_{z}$, $\hat{\Lambda }$, $\hat{\mu }$, $\hat{\Psi }$)) 10: value = ${\hat{\mu }}_{z}+{\hat{\Sigma }}_{z}{\hat{\Lambda }}^{⊤}{\left(\hat{\Lambda }{\hat{\Sigma }}_{z}{\hat{\Lambda }}^{⊤}+\hat{\Psi }\right)}^{-1}\left(x^{*}-\hat{\mu }-\hat{\Lambda }{\hat{\mu }}_{z}\right)$ 11: covarianceMatrix = ${\hat{\Sigma }}_{z}-{\hat{\Sigma }}_{z}{\hat{\Lambda }}^{⊤}{\left(\hat{\Lambda }{\hat{\Sigma }}_{z}{\hat{\Lambda }}^{⊤}+\hat{\Psi }\right)}^{-1}\hat{\Lambda }{\hat{\Sigma }}_{z}^{⊤}$ 12: **return** (value, covarianceMatrix)

**Algorithm A2** S2.FA—matrix form.
1: **function**Train(X,Z) ▹ $X=\left[\begin{array}{ccc} x^{\left(1\right)} & \ldots & x^{\left(n\right)} \end{array}\right]$ 2: ▹ $Z=\left[\begin{array}{ccc} z^{\left(1\right)} & \ldots & z^{\left(n\right)} \end{array}\right]$ 3: $\bar{x}=\frac{\sum_{i=1}^{n}X_{:i}}{n}$ 4: ${\hat{\mu }}_{z}=\frac{\sum_{i=1}^{n}Z_{:i}}{n}=\bar{z}$ 5: ${\hat{\Sigma }}_{z}=\frac{1}{n}\left(Z-{\hat{\mu }}_{z}\underset{\in R^{1\times n}}{\underset{︸}{\left[\begin{array}{cccc} 1 & 1 & \ldots & 1 \end{array}\right]}}\right){\left(Z-{\hat{\mu }}_{z}\left[\begin{array}{cccc} 1 & 1 & \ldots & 1 \end{array}\right]\right)}^{⊤}=\frac{1}{n}ZZ^{⊤}-{\hat{\mu }}_{z}{\hat{\mu }}_{z}^{⊤}$ ▹ 2 ways to compute 6: $\hat{\Lambda }=\left(n\bar{x}{\hat{\mu }}_{z}^{⊤}-XZ^{⊤}\right){\left(n{\hat{\mu }}_{z}{\hat{\mu }}_{z}^{⊤}-ZZ^{⊤}\right)}^{-1}$ 7: $\hat{\mu }=\bar{x}-\hat{\Lambda }\bar{z}$ 8: $\hat{\Psi }=\mathrm{diag}\left(\frac{1}{n}\left(X-\hat{\mu }\underset{\in R^{1\times n}}{\underset{︸}{\left[\begin{array}{cccc} 1 & 1 & \ldots & 1 \end{array}\right]}}-\hat{\Lambda }Z\right){\left(X-\hat{\mu }\left[\begin{array}{cccc} 1 & 1 & \ldots & 1 \end{array}\right]-\hat{\Lambda }Z\right)}^{⊤}\right)$ 9: **return** ( ${\hat{\mu }}_{z}$, ${\hat{\Sigma }}_{z}$, $\hat{\Lambda }$, $\hat{\mu }$, $\hat{\Psi }$) 10: **function**Test( $x^{*}$,( ${\hat{\mu }}_{z}$, ${\hat{\Sigma }}_{z}$, $\hat{\Lambda }$, $\hat{\mu }$, $\hat{\Psi }$)) 11: value = ${\hat{\mu }}_{z}+{\hat{\Sigma }}_{z}{\hat{\Lambda }}^{⊤}{\left(\hat{\Lambda }{\hat{\Sigma }}_{z}{\hat{\Lambda }}^{⊤}+\hat{\Psi }\right)}^{-1}\left(x^{*}-\hat{\mu }-\hat{\Lambda }{\hat{\mu }}_{z}\right)$ 12: covarianceMatrix = ${\hat{\Sigma }}_{z}-{\hat{\Sigma }}_{z}{\hat{\Lambda }}^{⊤}{\left(\hat{\Lambda }{\hat{\Sigma }}_{z}{\hat{\Lambda }}^{⊤}+\hat{\Psi }\right)}^{-1}\hat{\Lambda }{\hat{\Sigma }}_{z}^{⊤}$ 13: **return** (value, covarianceMatrix)

## Appendix C. S3.FA

The algorithms below are more general: *z* is not just a real number, as we state in the paper, but a real vector.

**Algorithm A3** S3.FA—nonmatrix form.
1: **function**LogLikelihood( $\left\{\left(x^{\left(1\right)},z^{\left(1\right)}\right),\ldots ,\left(x^{\left(a\right)},z^{\left(a\right)}\right),x^{\left(a+1\right)},\ldots ,x^{\left(n\right)}\right\}$, $\left(\mu_{z},\Sigma_{z},\mu ,\Lambda ,\Psi \right)$) 2: **return** $\sum_{i=1}^{a}\left(\ln\left(N(z^{\left(i\right)}|\mu_{z},\Sigma_{z})\right)+\ln\left(N(x^{\left(i\right)}|\mu +\Lambda z^{\left(i\right)},\Psi )\right)\right)+\sum_{i=a+1}^{n}\ln\left(N(x^{\left(i\right)}|\mu +\Lambda \mu_{z},\Lambda \Sigma_{z}\Lambda^{⊤}+\Psi )\right)$ 3: **function**Train( $\left\{\left(x^{\left(1\right)},z^{\left(1\right)}\right),\ldots ,\left(x^{\left(a\right)},z^{\left(a\right)}\right),x^{\left(a+1\right)},\ldots ,x^{\left(n\right)}\right\}$,nMaxIterations,eps) 4: $\bar{x}=\frac{\sum_{i=1}^{n}x^{\left(i\right)}}{n}$ 5: $\theta^{\left(0\right)}=\mathrm{initializeParameters}\left(\left\{\left(x^{\left(1\right)},z^{\left(1\right)}\right),\ldots ,\left(x^{\left(a\right)},z^{\left(a\right)}\right),x^{\left(a+1\right)},\ldots ,x^{\left(n\right)}\right\}\right)$ 6: $l_{\text{RV\_Do}}^{\left(0\right)}=$ LogLikelihood( $\left\{\left(x^{\left(1\right)},z^{\left(1\right)}\right),\ldots ,\left(x^{\left(a\right)},z^{\left(a\right)}\right),x^{\left(a+1\right)},\ldots ,x^{\left(n\right)}\right\}$, $\theta^{\left(0\right)}$) 7: **for** t = 0:nMaxIterations **do** 8: **E step**: Compute $E[Z^{\left(i\right)}]$, $E\left[Z^{\left(i\right)}{Z^{\left(i\right)}}^{⊤}\right],i\in \left\{a+1,\ldots ,n\right\}$: 9: $E_{Z^{\left(i\right)}|X^{\left(i\right)}=x^{\left(i\right)},\theta^{\left(t\right)}}\left[Z^{\left(i\right)}\right]=\mu_{z}^{\left(t\right)}+\Sigma_{z}^{\left(t\right)}{\Lambda^{\left(t\right)}}^{⊤}{\left(\Lambda^{\left(t\right)}\Sigma_{z}^{\left(t\right)}{\Lambda^{\left(t\right)}}^{⊤}+\Psi^{\left(t\right)}\right)}^{-1}\left(x^{\left(i\right)}-\mu^{\left(t\right)}\;-\Lambda^{\left(t\right)}\mu_{z}^{\left(t\right)}\right)$ 10: $E_{Z^{\left(i\right)}|X^{\left(i\right)}=x^{\left(i\right)},\theta^{\left(t\right)}}\left[Z^{\left(i\right)}{Z^{\left(i\right)}}^{⊤}\right]=\Sigma_{z}^{\left(t\right)}+E_{Z^{\left(i\right)}|X^{\left(i\right)}=x^{\left(i\right)},\theta^{\left(t\right)}}\left[Z^{\left(i\right)}\right]E_{Z^{\left(i\right)}|X^{\left(i\right)}=x^{\left(i\right)},\theta^{\left(t\right)}}{\left[Z^{\left(i\right)}\right]}^{⊤}$ 11: **M Step**: Compute $\theta^{\left(t+1\right)}=\left(\mu_{z}^{\left(t+1\right)},\Sigma_{z}^{\left(t+1\right)},\mu^{\left(t+1\right)},\Lambda^{\left(t+1\right)},\Psi^{\left(t+1\right)}\right)$: 12: $\mu_{z}^{\left(t+1\right)}=\frac{\sum_{i=1}^{a}z^{\left(i\right)}+\sum_{a+1}^{n}E\left[Z^{\left(i\right)}\right]}{n}$ 13: $\Sigma_{z}^{\left(t+1\right)}=\frac{1}{n}\left(\sum_{i=1}^{a}\left(z^{\left(i\right)}-\mu_{z}^{\left(t+1\right)}\right){\left(z^{\left(i\right)}-\mu_{z}^{\left(t+1\right)}\right)}^{⊤}+\sum_{i=a+1}^{n}\left(E\left[Z^{\left(i\right)}{Z^{\left(i\right)}}^{⊤}\right]\;-E\left[Z^{\left(i\right)}\right]{\mu_{z}^{\left(t+1\right)}}^{⊤}-\mu_{z}^{\left(t+1\right)}E{\left[Z^{\left(i\right)}\right]}^{⊤}+\mu_{z}^{\left(t+1\right)}{\mu_{z}^{\left(t+1\right)}}^{⊤}\right)\right)$ 14: $\Lambda^{\left(t+1\right)}=\left(n\bar{x}{\mu_{z}^{\left(t+1\right)}}^{⊤}-\sum_{i=1}^{a}x^{\left(i\right)}{z^{\left(i\right)}}^{⊤}-\sum_{i=a+1}^{n}x^{\left(i\right)}E{\left[Z^{\left(i\right)}\right]}^{⊤}\right)$ $\;{\left(n\mu_{z}^{\left(t+1\right)}{\mu_{z}^{\left(t+1\right)}}^{⊤}-\sum_{i=1}^{a}z^{\left(i\right)}{z^{\left(i\right)}}^{⊤}-\sum_{i=a+1}^{n}E\left[Z^{\left(i\right)}{Z^{\left(i\right)}}^{⊤}\right]\right)}^{-1}$ 15: $\mu^{\left(t+1\right)}=\bar{x}-\Lambda^{\left(t+1\right)}\mu_{z}^{\left(t+1\right)}$ 16: $\Psi^{\left(t+1\right)}=\mathrm{diag}\left(\frac{1}{n}\left(\sum_{i=1}^{a}\left(x^{\left(i\right)}-\mu^{\left(t+1\right)}-\Lambda^{\left(t+1\right)}z^{\left(i\right)}\right){\left(x^{\left(i\right)}-\mu^{\left(t+1\right)}-\Lambda^{\left(t+1\right)}z^{\left(i\right)}\right)}^{⊤}+\;\sum_{i=a+1}^{n}\left(\left(x^{\left(i\right)}-\mu^{\left(t+1\right)}\right){\left(x^{\left(i\right)}-\mu^{\left(t+1\right)}\right)}^{⊤}-\left(x^{\left(i\right)}-\mu^{\left(t+1\right)}\right)E{\left[Z^{\left(i\right)}\right]}^{⊤}{\Lambda^{\left(t+1\right)}}^{⊤}-\;\Lambda^{\left(t+1\right)}E\left[Z^{\left(i\right)}\right]{\left(x^{\left(i\right)}-\mu^{\left(t+1\right)}\right)}^{⊤}+\Lambda^{\left(t+1\right)}E\left[Z^{\left(i\right)}{Z^{\left(i\right)}}^{⊤}\right]{\Lambda^{\left(t+1\right)}}^{⊤}\right)\right)\right)$ 17: $l_{\text{RV\_Do}}^{\left(t+1\right)}=$ LogLikelihood( $\left\{\left(x^{\left(1\right)},z^{\left(1\right)}\right),\ldots ,\left(x^{\left(a\right)},z^{\left(a\right)}\right),x^{\left(a+1\right)},\ldots ,x^{\left(n\right)}\right\}$, $\theta^{\left(t+1\right)}$) 18: **if** $\frac{{∥\theta^{\left(t\right)}-\theta^{\left(t+1\right)}∥}_{2}^{2}}{{∥\theta^{\left(t\right)}∥}_{2}^{2}}\leq$eps **or** $\frac{|l_{\text{RV\_Do}}^{\left(t\right)}-l_{\text{RV\_Do}}^{\left(t+1\right)}|}{|l_{\text{RV\_Do}}^{\left(t\right)}|}\leq$eps **then** 19: **break** 20: **return** $\theta^{\left(t\right)}$ 21: **function**Test( $x^{*}$,( ${\hat{\mu }}_{z}$, ${\hat{\Sigma }}_{z}$, $\hat{\Lambda }$, $\hat{\mu }$, $\hat{\Psi }$)) ▹ The same as in *S2.FA* 22: value = ${\hat{\mu }}_{z}+{\hat{\Sigma }}_{z}{\hat{\Lambda }}^{⊤}{\left(\hat{\Lambda }{\hat{\Sigma }}_{z}{\hat{\Lambda }}^{⊤}+\hat{\Psi }\right)}^{-1}\left(x^{*}-\hat{\mu }-\hat{\Lambda }{\hat{\mu }}_{z}\right)$ 23: covarianceMatrix = ${\hat{\Sigma }}_{z}-{\hat{\Sigma }}_{z}{\hat{\Lambda }}^{⊤}{\left(\hat{\Lambda }{\hat{\Sigma }}_{z}{\hat{\Lambda }}^{⊤}+\hat{\Psi }\right)}^{-1}\hat{\Lambda }{\hat{\Sigma }}_{z}^{⊤}$ 24: **return** (value, covarianceMatrix)

**Algorithm A4** S3.FA—matrix form.
1: **function**LogLikelihood(X,Z, $\left(\mu_{z},\Sigma_{z},\mu ,\Lambda ,\Psi \right)$) ▹ $X=\left[\begin{array}{ccc} x^{\left(1\right)} & \ldots & x^{\left(n\right)} \end{array}\right]$ 2: ▹ $Z=\left[\begin{array}{ccc} z^{\left(1\right)} & \ldots & z^{\left(a\right)} \end{array}\right]$ 3: **return** $\sum_{i=1}^{a}\left(\ln\left(N(Z_{:i}|\mu_{z},\Sigma_{z})\right)+\ln\left(N(X_{:i}|\mu +\Lambda Z_{:i},\Psi )\right)\right)+\sum_{i=a+1}^{n}\ln\left(N(X_{:i}|\mu +\Lambda \mu_{z},\Lambda \Sigma_{z}\Lambda^{⊤}+\Psi )\right)$ 4: **function**Train(X,Z,nMaxIterations,eps) ▹ $X=\left[\begin{array}{ccc} x^{\left(1\right)} & \ldots & x^{\left(n\right)} \end{array}\right]$ 5: ▹ $Z=\left[\begin{array}{ccc} z^{\left(1\right)} & \ldots & z^{\left(a\right)} \end{array}\right]$ 6: $\bar{x}=\frac{\sum_{i=1}^{n}X_{:i}}{n}$ 7: $\theta^{\left(0\right)}=\mathrm{initializeParameters}\left(X,Z\right)$ 8: $l_{\text{RV\_Do}}^{\left(0\right)}=$ LogLikelihood(X,Z, $\theta^{\left(0\right)}$) 9: **for** t = 0:nMaxIterations **do** 10: **E step**: Compute $E[Z^{\left(i\right)}]$, $E\left[Z^{\left(i\right)}{Z^{\left(i\right)}}^{⊤}\right],i\in \left\{a+1,\ldots ,n\right\}$: 11: $E\_Z=\mu_{z}^{\left(t\right)}+\Sigma_{z}^{\left(t\right)}{\Lambda^{\left(t\right)}}^{⊤}{\left(\Lambda^{\left(t\right)}\Sigma_{z}^{\left(t\right)}{\Lambda^{\left(t\right)}}^{⊤}+\Psi^{\left(t\right)}\right)}^{-1}\left(X_{:,(a+1):n}-\mu^{\left(t\right)}\underset{\in R^{1\times (n-a)}}{\underset{︸}{\left[\begin{array}{cccc} 1 & 1 & \ldots & 1 \end{array}\right]}}-\Lambda^{\left(t\right)}\mu_{z}^{\left(t\right)}\underset{\in R^{1\times (n-a)}}{\underset{︸}{\left[\begin{array}{cccc} 1 & 1 & \ldots & 1 \end{array}\right]}}\right)$ 12: $E\_Z\_Z\_T=\Sigma_{z}^{\left(t\right)}+E\_ZE\_Z^{⊤}$ 13: $E\_Z=\left[\begin{array}{cc} Z & E\_Z \end{array}\right]$ 14: $E\_Z\_Z\_T=ZZ^{⊤}+E\_Z\_Z\_T$ 15: **M Step**: Compute $\theta^{\left(t+1\right)}=\left(\mu_{z}^{\left(t+1\right)},\Sigma_{z}^{\left(t+1\right)},\mu^{\left(t+1\right)},\Lambda^{\left(t+1\right)},\Psi^{\left(t+1\right)}\right)$: 16: $\mu_{z}^{\left(t+1\right)}=\frac{\sum_{i=1}^{n}E\_Z_{:i}}{n}$ 17: $\Sigma_{z}^{\left(t+1\right)}=\frac{1}{n}E\_Z\_Z\_T-\mu_{z}^{\left(t+1\right)}{\mu_{z}^{\left(t+1\right)}}^{⊤}$ 18: $\Lambda^{\left(t+1\right)}=\left(n\bar{x}{\mu_{z}^{\left(t+1\right)}}^{⊤}-XE\_Z^{⊤}\right)$ ${\left(n\mu_{z}^{\left(t+1\right)}{\mu_{z}^{\left(t+1\right)}}^{⊤}-E\_Z\_Z\_T\right)}^{-1}$ 19: $\mu^{\left(t+1\right)}=\bar{x}-\Lambda^{\left(t+1\right)}\mu_{z}^{\left(t+1\right)}$ 20: $\Psi^{\left(t+1\right)}=\mathrm{diag}\left(\frac{1}{n}\left(\left(X-\mu^{\left(t+1\right)}\underset{\in R^{1\times n}}{\underset{︸}{\left[\begin{array}{cccc} 1 & 1 & \ldots & 1 \end{array}\right]}}\right){\left(X-\mu^{\left(t+1\right)}\underset{\in R^{1\times n}}{\underset{︸}{\left[\begin{array}{cccc} 1 & 1 & \ldots & 1 \end{array}\right]}}\right)}^{⊤}-\;\left(X-\mu^{\left(t+1\right)}\underset{\in R^{1\times n}}{\underset{︸}{\left[\begin{array}{cccc} 1 & 1 & \ldots & 1 \end{array}\right]}}\right)E\_Z^{⊤}{\Lambda^{\left(t+1\right)}}^{⊤}-\Lambda^{\left(t+1\right)}E\_Z{\left(X-\mu^{\left(t+1\right)}\underset{\in R^{1\times n}}{\underset{︸}{\left[\begin{array}{cccc} 1 & 1 & \ldots & 1 \end{array}\right]}}\right)}^{⊤}+\;\Lambda^{\left(t+1\right)}E\_Z\_Z\_T{\Lambda^{\left(t+1\right)}}^{⊤}\right)\right)$ 21: $l_{\text{RV\_Do}}^{\left(t+1\right)}=$ LogLikelihood(X,Z, $\theta^{\left(t+1\right)}$) 22: **if** $\frac{{∥\theta^{\left(t\right)}-\theta^{\left(t+1\right)}∥}_{2}^{2}}{{∥\theta^{\left(t\right)}∥}_{2}^{2}}\leq$eps **or** $\frac{|l_{\text{RV\_Do}}^{\left(t\right)}-l_{\text{RV\_Do}}^{\left(t+1\right)}|}{|l_{\text{RV\_Do}}^{\left(t\right)}|}\leq$eps **then** 23: **break** 24: **return** $\theta^{\left(t\right)}$ 25: **function**Test( $x^{*}$,( ${\hat{\mu }}_{z}$, ${\hat{\Sigma }}_{z}$, $\hat{\Lambda }$, $\hat{\mu }$, $\hat{\Psi }$)) ▹ The same as in *S2.FA* 26: value = ${\hat{\mu }}_{z}+{\hat{\Sigma }}_{z}{\hat{\Lambda }}^{⊤}{\left(\hat{\Lambda }{\hat{\Sigma }}_{z}{\hat{\Lambda }}^{⊤}+\hat{\Psi }\right)}^{-1}\left(x^{*}-\hat{\mu }-\hat{\Lambda }{\hat{\mu }}_{z}\right)$ 27: covarianceMatrix = ${\hat{\Sigma }}_{z}-{\hat{\Sigma }}_{z}{\hat{\Lambda }}^{⊤}{\left(\hat{\Lambda }{\hat{\Sigma }}_{z}{\hat{\Lambda }}^{⊤}+\hat{\Psi }\right)}^{-1}\hat{\Lambda }{\hat{\Sigma }}_{z}^{⊤}$ 28: **return** (value, covarianceMatrix)

## Appendix D. MS3.FA

The algorithms below are more general: *z* is not just a real number, as we state in the paper, but a real vector.

**Algorithm A5** MS3.FA—Other functions.
1: **function**CondNormalNA(y,( μ, Σ)) 2: I = $\left\{i|y_{i}=NA\right\}$ ▹ I = Indexes 3: OI = $\left\{i|y_{i}\neq NA\right\}$ ▹ OI = Other Indexes 4: mean = $\mu_{I}+\Sigma_{I,OI}\Sigma_{OI}^{-1}\left(y_{OI}-\mu_{OI}\right)$ 5: covarianceMatrix = $\Sigma_{I}-\Sigma_{I,OI}\Sigma_{OI}^{-1}\Sigma_{I,OI}^{⊤}$ 6: $E{\left[Y\right]}_{I}$ = mean 7: $E{\left[Y\right]}_{OI}=y_{OI}$ 8: $E\left[YY^{⊤}\right]=E\left[Y\right]E{\left[Y\right]}^{⊤}$ 9: $E{\left[YY^{⊤}\right]}_{I,I}=$ $E{\left[YY^{⊤}\right]}_{I,I}$ + covarianceMatrix 10: **return** ( E[Y], $E[YY^{⊤}]$) 11: **function**FullNormal( $\mu_{z},\Sigma_{z},\mu ,\Lambda ,\Psi$) 12: mean = $\left[\begin{array}{c} \mu +\Lambda \mu_{z}\\ \mu_{z} \end{array}\right]$ 13: covarianceMatrix = $\left[\begin{array}{cc} \Lambda \Sigma_{z}\Lambda^{⊤}+\Psi & \Lambda \Sigma_{z}\\ {\left(\Lambda \Sigma_{z}\right)}^{⊤} & \Sigma_{z} \end{array}\right]$ 14: **return** (mean,covarianceMatrix) 15: **function**LogLikelihood( $\left\{\left(x^{\left(1\right)},z^{\left(1\right)}\right),\ldots ,\left(x^{\left(n\right)},z^{\left(n\right)}\right)\right\}$, ( $\mu_{z},\Sigma_{z},\mu ,\Lambda ,\Psi$)) 16: (mean,cov) = FullNormal( $\mu_{z},\Sigma_{z},\mu ,\Lambda ,\Psi$) 17: result = 0 18: **for** i = 1:n **do** 19: $y^{\left(i\right)}=\left[\begin{array}{c} x^{\left(i\right)}\\ z^{\left(i\right)} \end{array}\right]$ 20: OI = $\left\{j|y_{j}^{\left(i\right)}\neq NA\right\}$ ▹ OI = Other indexes 21: result = result + $\ln N(y_{OI}^{\left(i\right)}|{\mathrm{mean}}_{OI},{\mathrm{cov}}_{OI,OI})$ 22: **return** result

**Algorithm A6** MS3.FA—Train.
1: **function**Train( $\left\{\left(x^{\left(1\right)},z^{\left(1\right)}\right),\ldots ,\left(x^{\left(n\right)},z^{\left(n\right)}\right)\right\}$,nMaxIterations,eps) ▹ data can have NAs 2: *θ*^(0)^ = initializeParameters$\left(\left\{\left(x^{\left(1\right)},z^{\left(1\right)}\right),\ldots ,\left(x^{\left(n\right)},z^{\left(n\right)}\right)\right\}\right)$ 3: $l_{\text{RV\_Do}}^{\left(0\right)}=$ LogLikelihood $\left(\left\{\left(x^{\left(1\right)},z^{\left(1\right)}\right),\ldots ,\left(x^{\left(n\right)},z^{\left(n\right)}\right)\right\},\theta^{\left(0\right)}\right)$ 4: **for** t = 0:nMaxIterations **do** 5: **E step**: Compute the following for all i∈{1,…,n}: 6: $y^{\left(i\right)}=\left[\begin{array}{c} x^{\left(i\right)}\\ z^{\left(i\right)} \end{array}\right]$ 7: fullNormal = FullNormal$\left(\mu_{z}^{\left(t\right)},\Sigma_{z}^{\left(t\right)},\mu^{\left(t\right)},\Lambda^{\left(t\right)},\Psi^{\left(t\right)}\right)$ 8: $\left(E\left[Y^{\left(i\right)}\right],E\left[Y^{\left(i\right)}{Y^{\left(i\right)}}^{⊤}\right]\right)=$ CondNormalNA $\left(y^{\left(i\right)},\mathrm{fullNormal}\right)$ 9: $E\left[X^{\left(i\right)}\right]=E{\left[Y^{\left(i\right)}\right]}_{1:D}$ ▹ D = dim( $x^{\left(i\right)}$) 10: $E\left[Z^{\left(i\right)}\right]=E{\left[Y^{\left(i\right)}\right]}_{\left(D+1):(D+d\right)}$ ▹ d = dim( $z^{\left(i\right)}$) 11: $E\left[X^{\left(i\right)}{X^{\left(i\right)}}^{⊤}\right]=E{\left[Y^{\left(i\right)}{Y^{\left(i\right)}}^{⊤}\right]}_{1:D,1:D}$ 12: $E\left[X^{\left(i\right)}{Z^{\left(i\right)}}^{⊤}\right]=E{\left[Y^{\left(i\right)}{Y^{\left(i\right)}}^{⊤}\right]}_{1:D,(D+1):(D+d)}$ 13: $E\left[Z^{\left(i\right)}{X^{\left(i\right)}}^{⊤}\right]=E{\left[X^{\left(i\right)}{Z^{\left(i\right)}}^{⊤}\right]}^{⊤}$ 14: $E\left[Z^{\left(i\right)}{Z^{\left(i\right)}}^{⊤}\right]=E{\left[Y^{\left(i\right)}{Y^{\left(i\right)}}^{⊤}\right]}_{\left(D+1):(D+d),(D+1):(D+d\right)}$ 15: **M Step**: Compute $\theta^{\left(t+1\right)}=\left(\mu_{z}^{\left(t+1\right)},\Sigma_{z}^{\left(t+1\right)},\mu^{\left(t+1\right)},\Lambda^{\left(t+1\right)},\Psi^{\left(t+1\right)}\right)$: 16: $\mu_{z}^{\left(t+1\right)}=\frac{\sum_{i=1}^{n}E\left[Z^{\left(i\right)}\right]}{n}$ 17: $\Sigma_{z}^{\left(t+1\right)}=\frac{\sum_{i=1}^{n}\left(E\left[Z^{\left(i\right)}{Z^{\left(i\right)}}^{⊤}\right]-E\left[Z^{\left(i\right)}\right]{\mu_{z}^{\left(t+1\right)}}^{⊤}-\mu_{z}^{\left(t+1\right)}E\left[{Z^{\left(i\right)}}^{⊤}\right]+\mu_{z}^{\left(t+1\right)}{\mu_{z}^{\left(t+1\right)}}^{⊤}\right)}{n}$ 18: $\bar{x}=\frac{\sum_{i=1}^{n}E\left[X^{\left(i\right)}\right]}{n}$ 19: $\Lambda^{\left(t+1\right)}=\left(n\bar{x}{\mu_{z}^{\left(t+1\right)}}^{⊤}-\sum_{i=1}^{n}E\left[X^{\left(i\right)}{Z^{\left(i\right)}}^{⊤}\right]\right){\left(n\mu_{z}^{\left(t+1\right)}{\mu_{z}^{\left(t+1\right)}}^{⊤}-\sum_{i=1}^{n}E\left[Z^{\left(i\right)}{Z^{\left(i\right)}}^{⊤}\right]\right)}^{-1}$ 20: $\mu^{\left(t+1\right)}=\bar{x}-\Lambda^{\left(t+1\right)}\mu_{z}^{\left(t+1\right)}$ 21: $\Psi^{\left(t+1\right)}=\mathrm{diag}\left(\frac{1}{n}\sum_{i=1}^{n}\left(E\left[X^{\left(i\right)}{X^{\left(i\right)}}^{⊤}\right]-E\left[X^{\left(i\right)}\right]{\mu^{\left(t+1\right)}}^{⊤}-\mu^{\left(t+1\right)}E{\left[X^{\left(i\right)}\right]}^{⊤}+\;\mu^{\left(t+1\right)}{\mu^{\left(t+1\right)}}^{⊤}-E\left[X^{\left(i\right)}{Z^{\left(i\right)}}^{⊤}\right]{\Lambda^{\left(t+1\right)}}^{⊤}+\mu^{\left(t+1\right)}E{\left[Z^{\left(i\right)}\right]}^{⊤}{\Lambda^{\left(t+1\right)}}^{⊤}-\Lambda^{\left(t+1\right)}E\left[Z^{\left(i\right)}{X^{\left(i\right)}}^{⊤}\right]+\;\Lambda^{\left(t+1\right)}E{\left[Z^{\left(i\right)}\right]}^{⊤}{\mu^{\left(t+1\right)}}^{⊤}+\Lambda^{\left(t+1\right)}E\left[Z^{\left(i\right)}{Z^{\left(i\right)}}^{⊤}\right]{\Lambda^{\left(t+1\right)}}^{⊤}\right)\right)$ 22: $l_{\text{RV\_Do}}^{\left(t+1\right)}$ = LogLikelihood $\left(\left\{\left(x^{\left(1\right)},z^{\left(1\right)}\right),\ldots ,\left(x^{\left(n\right)},z^{\left(n\right)}\right)\right\},\theta^{\left(t+1\right)}\right)$ 23: **if** $\frac{{∥\theta^{\left(t\right)}-\theta^{\left(t+1\right)}∥}_{2}^{2}}{{∥\theta^{\left(t\right)}∥}_{2}^{2}}\leq$eps **or** $\frac{|l_{\text{RV\_Do}}^{\left(t\right)}-l_{\text{RV\_Do}}^{\left(t+1\right)}|}{|l_{\text{RV\_Do}}^{\left(t\right)}|}\leq$eps **then** 24: **break** 25: **return** $\theta^{\left(t\right)}$

**Algorithm A7** MS3.FA—Test and Impute.
1: **function**Test( $y^{*}=\left(x^{*},z^{*}\right)$ partially known, ( ${\hat{\mu }}_{z}$, ${\hat{\Sigma }}_{z}$, $\hat{\Lambda }$, $\hat{\mu }$, $\hat{\Psi }$)) 2: fullNormal = FullNormal$\left({\hat{\mu }}_{z},{\hat{\Sigma }}_{z},\hat{\mu },\hat{\Lambda },\hat{\Psi }\right)$ 3: $\left(E\left[Y^{*}\right],E\left[Y^{*}{Y^{*}}^{⊤}\right]\right)=$ CondNormalNA(*y**, fullNormal) 4: value = $E[Y^{*}]$ 5: covarianceMatrix = $E\left[Y^{*}{Y^{*}}^{⊤}\right]-E\left[Y^{*}\right]E{\left[Y^{*}\right]}^{⊤}$ 6: **return** (value,covarianceMatrix) 7: **function**Impute( $y^{*}=\left(x^{*},z^{*}\right)$ partially known, ( ${\hat{\mu }}_{z}$, ${\hat{\Sigma }}_{z}$, $\hat{\Lambda }$, $\hat{\mu }$, $\hat{\Psi }$)) 8: (value, covarianceMatrix) = Test( $y^{*}$,( ${\hat{\mu }}_{z}$, ${\hat{\Sigma }}_{z}$, $\hat{\Lambda }$, $\hat{\mu }$, $\hat{\Psi }$)) 9: **return** value
